# Wheat potassium transporter TaHAK13 mediates K^+^ absorption and maintains potassium homeostasis under low potassium stress

**DOI:** 10.3389/fpls.2022.1103235

**Published:** 2022-12-23

**Authors:** Yonghang Run, Xiyong Cheng, Wei Dou, Yue Dong, Yanan Zhang, Bingbing Li, Tengfei Liu, Haixia Xu

**Affiliations:** National Key Laboratory of Wheat and Maize Crop Science/Agronomy College, Henan Agricultural University, Zhengzhou, China

**Keywords:** Wheat (*Triticum aestivum* L.), *TaHAK13*, Low potassium stress, function characterization, interaction protein

## Abstract

Potassium (K) is an essential nutrient for plant physiological processes. Members of the HAK/KUP/KT gene family act as potassium transporters, and the family plays an important role in potassium uptake and utilization in plants. In this study, the *TaHAK13* gene was cloned from wheat and its function characterized. Real-time quantitative PCR (RT-qPCR) revealed that *TaHAK13* expression was induced by environmental stress and up-regulated under drought (PEG6000), low potassium (LK), and salt (NaCl) stress. GUS staining indicated that *TaHAK13* was mainly expressed in the leaf veins, stems, and root tips in *Arabidopsis thaliana*, and expression varied with developmental stage. *TaHAK13* mediated K^+^ absorption when heterologously expressed in yeast CY162 strains, and its activity was slightly stronger than that of a *TaHAK1* positive control. Subcellular localization analysis illustrated that TaHAK13 was located to the plasma membrane. When c(K^+^) ≤0.01 mM, the root length and fresh weight of *TaHAK13* transgenic lines (*athak5/TaHAK13*, Col*/TaHAK13*) were significantly higher than those of non-transgenic lines (*athak5*, Col). Non-invasive micro-test technology (NMT) indicated that the net K influx of the transgenic lines was also higher than that of the non-transgenic lines. This suggests that *TaHAK13* promotes K^+^ absorption, especially in low potassium media. Membrane-based yeast two-hybrid (MbY2H) and luciferase complementation assays (LCA) showed that TaHAK13 interacted with TaNPF5.10 and TaNPF6.3. Our findings have helped to clarify the biological functions of TaHAK13 and established a theoretical framework to dissect its function in wheat.

## 1 Introduction

Potassium ions (K^+^) are the most abundant cation in plants and are involved in many physiological and biochemical processes, including cell elongation, enzyme activity regulation, osmotic regulation, stomatal movement, photosynthesis, and protein synthesis (Zhang et al., 2012). Potassium also acts as a transporter for photosynthetic substances (from source to sink) and participates in the regulation of osmotic pressure and the plant response to osmotic stress (Gajdanowicz et al., 2011). A moderate increase in potassium application rate is helpful for enhancing plant resistance to abiotic stress (Wang et al., 2021). Potassium is also closely tied to crop quality (Song et al., 2014). The cytoplasmic concentration of K^+^ for normal growth in living cells is approximately 100 mM (40-200 mM), which is also the optimum concentration for normal enzymatic function. Compared to the higher concentrations of K^+^ observed in living cells, the concentration of potassium ions in the root-soil interface tends to be at the micromolar level (0.1-1 mM). In most cases, potassium uptake by plants is an active transport process against a concentration gradient (Maathuis, 2009). The absorption of K^+^ from the external environment into plant cells and its transport within plant tissues are mainly completed by K transporters and K channel proteins. These genes can be divided into five families according to the structure and function of K transporters and channel proteins, including three transporter families (KUP/HAK/KT, HKT, and CPA families) and two ion channel protein families (Shaker and KCO/TPK families). Transporters combine with K^+^, undergo conformational changes, and then transport K^+^ across the cell membrane. Channel proteins form water channels through the lipid bilayer that allow K^+^ to pass through the membrane when the channel is open (Wang et al., 2010).

The KUP/HAK/KT transporter family is present in bacteria, fungi, and plants, and participates in K^+^ transmembrane transport (Vastermark et al., 2014). Characterizing the physiological functions of the KUP/HAK/KT potassium transporter family in plants has recently been the focus of much research attention. There are 13 proteins belonging to the KUP/HAK/KT family in *Arabidopsis* (Mäser et al., 2001), 25 in rice (Yang et al., 2009), 5 in barley (Alemán et al., 2011), 27 in corn (Zhang et al., 2012), and 56 in wheat (Cheng et al., 2018). Proteins in the KUP/HAK/KT family are divided into four clusters: cluster I, cluster II, cluster III, and cluster IV. Members of cluster I have been extensively studied, including AtHAK5 (*Arabidopsis thaliana*), HvHAK1 (*Hordeum vulgare* L.), and OsHAK1/OsHAK5 (*Oryza sativa* L.). The HvHAK1 protein is localized in the plasma membrane and primarily expressed in roots; it mediates the high-affinity absorption of potassium ions and is strongly induced by low potassium conditions (Senn et al., 2001). In *Arabidopsis thaliana*, AtHAK5 is a member of the high affinity K^+^ absorption system, which is induced by potassium starvation (no K^+^ supply) and expressed in plant roots (Ahn et al., 2004; Armengaud et al., 2004; Shin and Schachtman, 2004). AtHAK5 maintains a very high level of expression after seven days of potassium starvation (Gierth et al., 2005). Under low potassium stress (< 50 μM K^+^), *athak5* mutant seeds germinate slowly, root elongation is inhibited, and the ability to absorb K^+^ decreases, indicating that AtHAK5 mediates the absorption of high affinity K^+^ and participates in the process of seed germination and later growth and development (Rubio et al., 2008; Pyo et al., 2010). The transcription of OsHAK5 increases during potassium starvation and under salt stress; cells accumulate a large amount of K^+^ (instead of Na^+^) when expressed in tobacco BY2 cells, suggesting that OsHAK5 is a salt-sensitive high affinity K^+^ transporter (Horie et al., 2011). In addition, OsHAK1 and OsHAK5 are the two main K^+^ transporters active in low potassium stress conditions; the transport activity of OsHAK1, unlike that of OsHAK5, is sensitive to Na^+^ (Okada et al., 2018). The K^+^ absorption rate and transport capacity of wild type plants is significantly higher than that of mutant plants with OsHAK1 gene knockout. Over-expression of OsHAK1 significantly enhances K^+^ absorption capacity, suggesting that this protein affects K^+^ absorption and may simultaneously mediate K^+^ absorption and transportation by the two K^+^ absorption systems (Chen et al., 2015; Chen et al., 2019). Finally, the PhaHAK5 protein was identified in a salt-sensitive reed and belongs to cluster IV. Functional analyses in yeast have found it acts as a high affinity potassium transporter, capable of mediating low affinity sodium ion transport in the presence of high Na^+^ stress (Takahashi et al., 2007).

A total of 56 HAK/KUP/KT family members (TaHAK1-TaHAK25, containing homologous genes) were identified in wheat in a recent phylogenetic analysis (Cheng et al., 2018). The HAK/KUP/KT family includes vital transporter proteins for potassium homeostasis, but very little is known as to the detailed functions of these proteins in plants. In this study, *TaHAK13* was investigated in a physiological function analysis to better understand the molecular mechanisms underlying efficient K^+^ transport in wheat.

## 2 Materials and methods

### 2.1 Plant materials and growth conditions

Hexaploid wheat (*Triticum aestivum* L., cv. Yunong 804) seeds were germinated in the dark at 25°C for two days after sterilization; seeds were sprayed daily with ddH_2_O to keep them moist. After five days, seedlings were transplanted into Hoagland nutrient solution (**Table S1**), and this solution was changed every three days. Two-week-old wheat seedlings were divided into four groups, with each group containing at least 30 plantlets. The four groups were: a control (Hoagland medium with full K^+^ concentration of 1mM KCl), a drought treatment (20% PEG6000+Hoagland medium), a low potassium treatment (K_2_SO_4_ concentration in Hoagland medium of 0.01 mM), and a salt stress treatment (200 mM NaCl + Hoagland medium). Plantlets were maintained on the medium at 25°C and a 16 h light/8 h dark photoperiod. Seedling roots were sampled at the following time points: 0, 1, 3, 6, 9, 12, and 24 h. Each sample was immediately frozen in liquid nitrogen and stored at -80°C for further analyses.

Tobacco seeds (*Nicotiana benthamiana*) were sown on nutrient soil and kept in a growth chamber at 25°C and 50-70% relative humidity with a photoperiod of 16 h light/8 h dark for about four weeks; these were used for the assessment of *Agrobacterium*-mediated transient fluorescent protein fusion expression.

Wild-type (Col-0) and mutant (*athak5*, SALK_005604) *Arabidopsis* seeds were surface sterilized with 70% (v/v) ethanol for 7 min and 0.1% (v/v) NaClO for 5 min, then washed with ddH_2_O three times. Sterilized seeds were stored at 4°C in the dark for three days to promote synchronous germination. Phenotypic analysis of seedlings was carried out under low potassium conditions on Murashige Skoog (MS) medium following protocols described by Pyo et al. (Pyo et al., 2010). For soil culture, plants were pre-cultured in MS basic medium for seven days, then transferred to nutrient soil and cultured in a growth chamber with a photoperiod of 16 h light/8 h dark at 25°C.

### 2.2 Real-time quantitative PCR (RT-qPCR)

Total RNA was extracted from all samples using Trizol (TransGen Biotech) according to the manufacturer’s instructions. First strand cDNA was synthesized using a PrimeScript™ RT reagent Kit with a gDNA Eraser (Takara). The diluted cDNA was amplified using qPCR SYBR Green Master Mix (Yeasen) on a real-time PCR system (Quantstudio™5) following standard protocols. The primer sequences used are listed in **Table S2**. The qPCR procedures were as follows: 95°C for 5 min, followed by 40 cycles of 95°C for 15 s and 61°C for 1 min, and 72°C for 5 min. Three biological replicates were used in each independent experiment and three independent experiments were performed for each RT-qPCR data analysis. Relative transcript levels were calculated using the 2^−ΔΔCt^ method with the wheat *β*-*actin* gene as an internal reference control (Livaka and Schmittgen, 2001).

### 2.3 Isolation of the *TaHAK13* gene and subcellular localization analysis

The coding sequence (CDS) of the *TaHAK13* gene (2,412 bp) was amplified from the cDNA isolated from wheat seedlings. Sequence data for *TaHAK13* (ID: TraesCS7D02G456900) were obtained from the wheat genome annotation project (Ensembl Plants database). The primer sequence used for amplifying the coding sequence is provided in **Table S2**. PCR products were cloned into a pESI-Blunt vector using the Zero TOPO-TA Cloning Kit (Yeasen) and then sequenced.

The coding sequence of *TaHAK13*, which contains *Sac*I and *Bam*HI restriction sites without a stop codon, was amplified and inserted in front of the GFP gene sequence in a 35S-GFP vector. The TaHAK13-GFP fusion vector, under the control of a 35S promoter, was then transformed into *Agrobacterium tumefaciens* strain GV3101. Positive strains were injected into tobacco leaf epidermal cells using the agroinfiltration method. Before imaging, transformed plants were grown for two days at 22^°^C with a 16 h light/8 h dark photoperiod. The GFP signal was visualized with a confocal laser-scanning microscope (Carl Zeiss, Germany). The primer sequences used are given in **Table S2**.

### 2.4 GUS staining assay

A 2,250 bp fragment was cloned from upstream of the *TaHAK13* start codon in wheat genomic DNA using relevant primers (**Table S2**) with a restriction site. The amplified DNA fragment used a *TaHAK13* promoter, replacing the LacZ and CaMV35S promoter regions, and were constructed in a pCAMBIA1304 vector. The constructed plasmid was transformed into *Agrobacterium tumefaciens* GV3101 to infect *Arabidopsis via* the flower soaking method. The T_2_ generation plants were used for GUS histochemical staining. Using previously published methods (Jefferson et al., 1987), the roots, leaves, stems, flowers, pods, and other tissues of both *Arabidopsis* seedlings and mature plants were stained with a GUS staining kit (Coolaber). The colored part was observed and photographed using a stereomicroscope (Stemi508, Carl Zeiss, Germany).

### 2.5 Functional complementation of *TaHAK13* in the yeast strain CY162

*TaHAK13* was amplified with specific primers (**Table S2**), digested with *Bam*HI or *Xba*I restriction endonucleases, and ligated to the yeast expression vector p416. In a functional complementation experiment, p416, *TaHAK13*-p416, and *TaHAK1*-p416 were transformed into yeast strains CY162 (*MATα, ura3, his3, ade2, trk1△, trk2△::PCK64*) (Anderson et al., 1992) and AXT3K (*MATα, ena1::HIS3::ena4, nha1::LEU2, nhx1::KanMX4*), respectively (Quintero et al., 2000).

The yeast complementation analysis was carried out on solid AP-U medium (i.e., an arginine phosphate medium lacking uracil) (Xu et al., 2008), with a supplemental K^+^ concentration in the range of 0-100 mM. The experimental yeast strains (transformed with p416, *TaHAK13*-p416, or *TaHAK1*-p416) were grown overnight in liquid SD-U medium (i.e., a synthetic defined base without uracil) and then transferred to liquid AP-U medium supplemented with different concentrations of K^+^ (either 1 mM or 100 mM) with the same initial OD_600_ (~0.001). Once in the AP-U medium, the yeast strains were grown for three days on a shaker at 220 r/min. The OD_600_ of each strain was measured every 8 h. The experiment was repeated three times.

### 2.6 K^+^ depletion and K^+^ content determination of the yeast strain CY162

A K^+^ depletion experiment was performed according to previously described procedures (Zhang et al., 2018) with minor modifications. Yeast cells (transformed with p416, *TaHAK13*-p416, or *TaHAK1*-p416) were grown overnight in liquid SD-U medium at 30°C, and then transferred to AP-U liquid medium for potassium starvation for about 4 h. Cells were suspended in 10 mM MES supplemented with 2% glucose and adjusted to pH 6.0 with Ca(OH)_2_. At time zero, KCl was added to the culture medium, and samples were collected every 20 min within 2 h.

To measure the K^+^ content in yeast cells, yeast strains (transformed with p416, *TaHAK13*-p416, or *TaHAK1*-p416) were grown in AP-U medium with different K^+^ concentrations (0-100 mM) at 30°C. Yeast cells were first suspended in pre-cooled sterile water with an OD_600_ = 0.3, and then repeatedly heated and frozen to break the cells. A flame photometer (FP640) was used to measure the K^+^ content. The specific operation method was as described by Karabegov (Karabegov, 2011). Three replicates from each sample were tested in total.

### 2.7 *TaHAK13* gene expression in wild type (Col) and mutant (*athak5*) *Arabidopsis*

The *TaHAK13* coding sequence was amplified and used to construct a pCAMBIA1300 vector utilizing a CaMV35S promoter with kanamycin resistance. The resulting plasmid was introduced into *Agrobacterium tumefaciens* strain GV3101 for transformation into mutant (*athak5*) and wild-type (Col) *Arabidopsis* using the floral dip method. Transgenic seedlings (Col/*TaHAK13* and *athak5*/*TaHAK13*) were confirmed by RT-PCR. Transgenic lines and non-transgenic lines were then planted in MS medium with different K^+^ concentrations (0, 0.01, 0.1, and 1 mM KCl), and phenotypes (root length and fresh weight) of transgenic and non-transgenic lines measured after ten days.

### 2.8 Determination of the net K^+^ influx in transgenic *Arabidopsis* roots

A non-invasive micro-test technology (NMT) system NMT100-SIM-XY (Younger USA Science and Technology; Xuyue, China) was used to determine the net K^+^ influx in transgenic *Arabidopsis* roots, and NMT User Manual 4.1 was referenced for specific operation methods. *Arabidopsis* seedlings were grown on MS medium for 10 d and then treated with a low potassium solution for 12 h. Before measuring the K^+^ influx, the ion-selective electrode was calibrated with K^+^ concentrations of 0.05 mM, 0.1 mM, and 0.5 mM. To take measurements, seedling roots were soaked in a preparatory solution (0.1 mM CaCl_2_ and 0.3 mM MES, pH 6.0) for 30 min, before transferring to a measuring solution supplied with 0.1 mM KCl or 0.01 mM KCl. The net K^+^ influx was measured over the course of 8 min under experimental conditions to reduce variability caused by solution fluctuation. Under the microscope, measurement sites were located at 0 μm, 200 μm, 400 μm, and 600 μm away from the root tip, and a microsensor was placed at approximately 150 µm from the root tip to optimize data collection. Flux rates were calculated; note positive values represent efflux, and negative values represent influx. In a separate experiment, measurements were collected from the roots of at least eight *Arabidopsis* plants, and each plant was measured once.

### 2.9 Membrane-based yeast two-hybrid assay

A membrane-based yeast two-hybrid system was used to screen for proteins interacting with TaHAK13 (Stagljar et al., 1998). The coding sequence of *TaHAK13* was introduced into a pBT3-N vector (see **Table S2** for primer sequences); the pBT3-N vector was then used as a bait vector, and the bait vector and library plasmid were co-transformed into the yeast strain NMY51 (*MATα, his3Δ200, trp1-901, leu2-3, 112, ade2, LYS2::(lexAop)_4_-HIS3, ura3::(lexAop)_8_-lacZ, ade2::(lexAop)_8_-ADE2, GAL4*). Proteins interacting with TaHAK13 were identified and then ligated into a pPR3-N vector (**Table S2**). To assay different protein combinations, the bait vector and prey vector were co-transformed into the yeast NMY51 strain, which was then grown on screening media (SD/-L/-T, SD/-A/-H/-L/-T); proteins were identified using X-Gal after culturing at 30°C for 3 d.

### 2.10 Dual-luciferase complementation assay

A luciferase complementation assay was used to analyze protein-protein interactions between TaHAK13 and either TaNPF5.10 or TaNPF6.3 (Wang et al., 2020). The coding sequence of *TaHAK13* was inserted into a pCAMBIA1300-nLUC vector, and the full CDS of *TaNPF5.10* and *TaNPF6.3* were separately ligated into pCAMBIA1300-cLUC vectors. The *Agrobacterium* strain GV3101 carrying these vectors was infiltrated into four-week-old tobacco leaves using the *Agrobacterium*-mediated transient transformation method (Chen et al., 2008). After three days of infiltration, 1 mM D-fluorescein potassium salt (Yeason) was sprayed on the leaves, which were then kept in the dark for 10 min. The luciferase signal was captured using a plant living image system (Night SHADE LB 985, Berthold, Germany).

### 2.11 Statistical analysis

Three independent biological repeats were set for each experiment. All data were subjected to analysis of One-way ANOVA according to the model for completely randomized design *via* SPSS 24.0 software (USA) and represented as mean. Significant differences were calculated based on *t*-test at *P*<0.05 level between different treatments.

## 3 Results

### 3.1 Analysis of *TaHAK13* expression under different stresses

**Figure 1:**
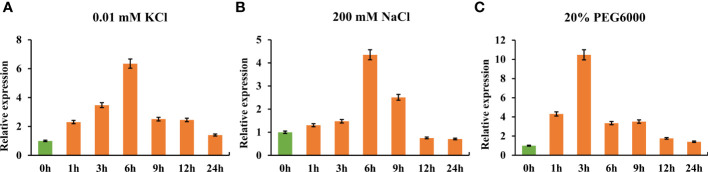
Real-time quantitative PCR expression of *TaHAK13* in wheat roots under different stresses. **(A)** The relative expression of *TaHAK13* under low potassium stress (0.01 mM KCl). **(B)** The relative expression of *TaHAK13* under salt stress (200 mM NaCl). **(C)** The relative expression of *TaHAK13* under dehydration stress (20% PEG6000). The relative expression of *TaHAK13* at 0 h was taken as a value of one.

To quantify *TaHAK13* expression under short-term environmental stress, RNA was extracted from the roots of wheat seedlings grown in low potassium, salt, and drought stress conditions. Using RT-qPCR, *TaHAK13* expression was then analyzed. Expression was first up-regulated and then down-regulated for all three stresses. In the low potassium (0.01 mM KCl) treatment, *TaHAK13* expression reached a maximum (six times that of the control) at 6 h, after then decreasing (**Figure 1A**). In the salt stress (200 mM NaCl) treatment, *TaHAK13* expression showed a similar pattern: up-regulation until the 6 h mark and then a gradual decline (**Figure 1B**). To determine whether the expression of *TaHAK13* was induced by dehydration, 15-day-old plantlets were transferred to a hydroponic solution supplemented with 20% PEG6000. The expression of *TaHAK13* was initially up-regulated, reaching a maximum (about ten times the 0 h value) at 3 h post-treatment (**Figure 1C**). This suggests that *TaHAK13* expression was induced by transient abiotic stress (**Figure 1** and **Figure S1**).

### 3.2 TaHAK13 was localized in the plasma membrane

**Figure 2:**
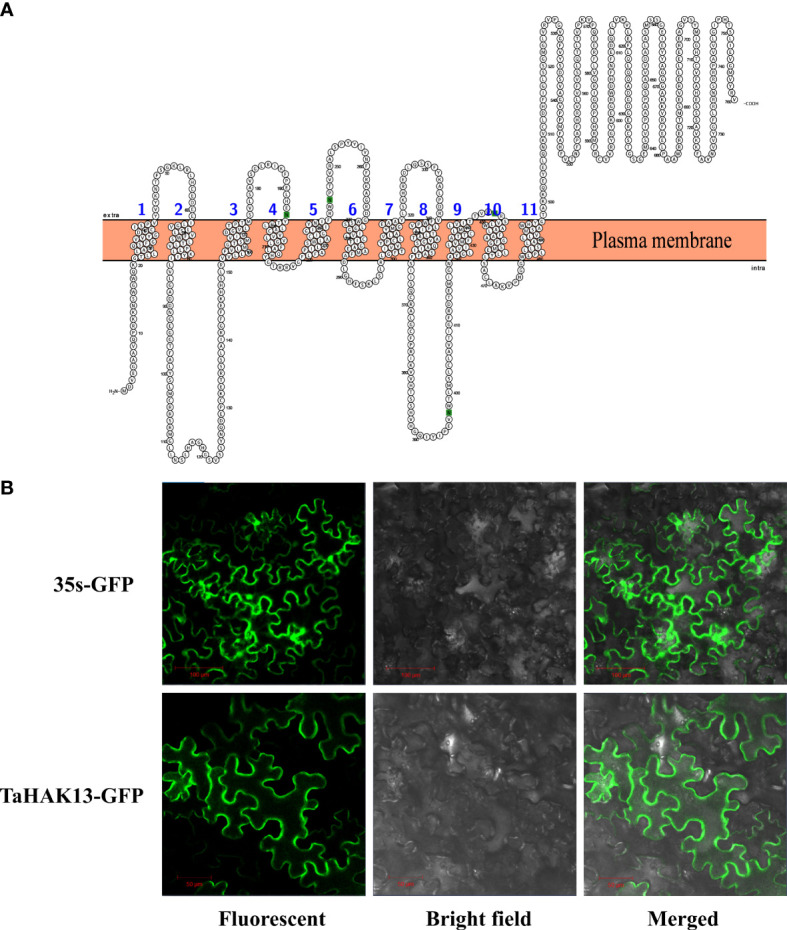
Characteristics of TaHAK13. **(A)** Predicted transmembrane domains of TaHAK13. **(B)** Subcellular localization of TaHAK13 in tobacco leaves. 35S-GFP acted as the control. Bar = 50 or 100 μm.

The transmembrane domain analysis identified eleven transmembrane structures in the TaHAK13 protein, with the N-terminal of the protein located in the cell membrane and the C-terminal outside the membrane (**Figure 2A**). The TaHAK13 protein may therefore play an important role in transmembrane transport. To further study the subcellular localization of TaHAK13, a vector containing a TaHAK13-GFP fusion protein was introduced into the epidermal cells of *Nicotiana benthamiana* leaves *via Agrobacterium tumefaciens* infection. The subcellular localization of the TaHAK13-GFP fusion protein and 35S-GFP (a control vector) was observed using a laser confocal microscope. The 35S-GFP control was expressed in the nucleus, cell membrane, and cytoplasm, while TaHAK13-GFP was expressed only in the plasma membrane (**Figure 2B**).

### 3.3 Expression specificity of *TaHAK13* in different tissues

**Figure 3:**
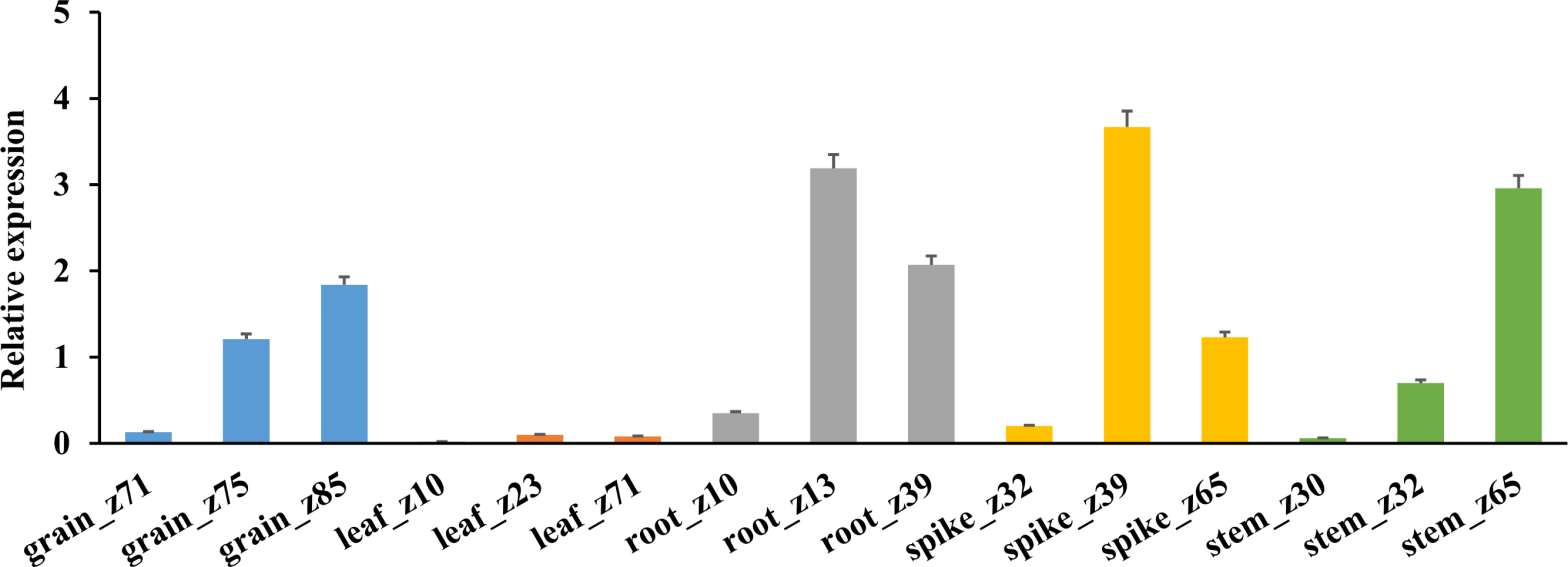
*TaHAK13* expression in different tissues. An RNA-seq analysis of wheat expression data was used to assess the expression specificity of *TaHAK13* in different tissues.

The expression specificity of *TaHAK13* in different tissues (roots, stems, leaves, spikes, and grains) was analyzed using RNA-Seq data from the wheat expression database (**Figure 3**). The average *TaHAK13* expression was highest in roots, followed by spikes and grains, and relatively low in stems; the lowest values were seen in leaves.

To study *TaHAK13* expression in greater detail, the 2,250 bp fragment upstream of the gene was cloned and used as a promoter. The promoter region of the *TaHAK13* gene contained a TATA-box, CAAT-box, stress response elements (e.g., an MYB binding site involved in drought resistance), *cis*-acting elements (involved in defense and stress responses), and a WRKY-binding W-box. Thus, the *TaHAK13* gene promoter is regulated by many factors. In addition, a key element (the Root motif TAPOX1) required for root specific expression was also found in the promoter, indicating that *TaHAK13* is highly expressed in roots (**Table S3**).

**Figure 4:**
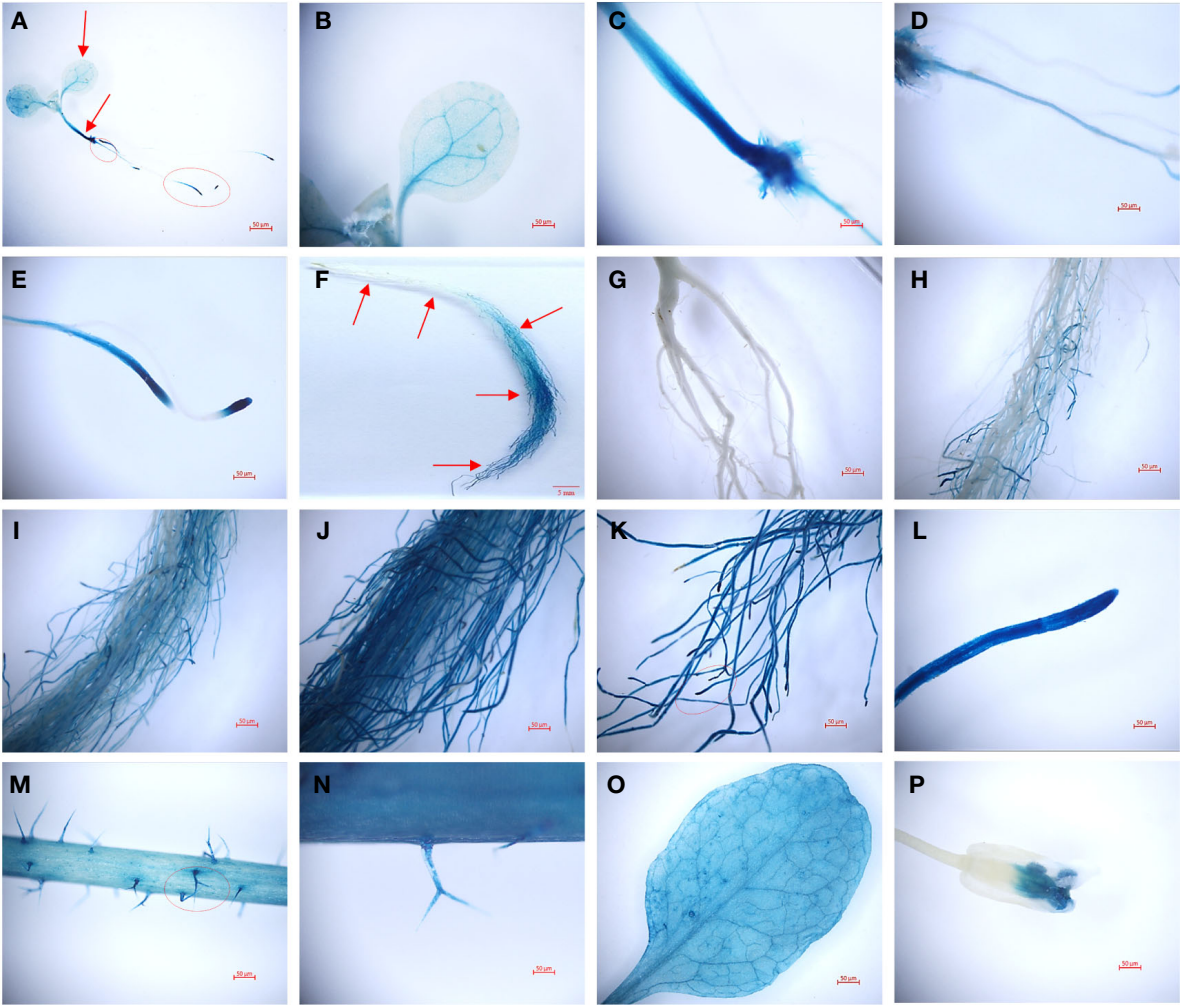
GUS histochemical staining with a *TaHAK13* promoter in transgenic *Arabidopsis thaliana*. **(A-E)** Zoomed out image of a transgenic *Arabidopsis* seedling **(A)** and its leaf **(B)**, stem **(C)**, taproot **(D)**, and lateral root apex **(E)**. **(F-P)** Zoomed out image of a root **(F)** and enlarged images of the indicated root positions (marked with red arrows) **(G-L)** in mature transgenic *Arabidopsis* plants; also pictured are the stem **(M)**, stem surface **(N)**, leaf **(O)**, and flower **(P)**.

Transgenic plants harboring a *TaHAK13* promoter-GUS fusion vector were used to investigate specific tissue expression patterns. Strong signals were detected in whole *Arabidopsis* plantlets in a GUS staining assay (**Figure 4**). The GUS gene (controlled by a *TaHAK13* promoter) was mainly expressed in the veins (**Figures 4A, B**), vascular bundle tissue of the embryonic axis (**Figure 4C**), taproot (**Figure 4D**), and lateral root apex (**Figure 4E**) of transgenic *Arabidopsis* seedlings. The highest *TaHAK13* specificity was observed in roots. To explore whether expression was sensitive to developmental stage, GUS staining was also carried out in the roots, leaves, stems, flowers, and pods of mature *Arabidopsis* plants. The veins (**Figure 4O**), roots (**Figure 4F**), and stems (**Figure 4M**) of transgenic mature *Arabidopsis* plants were deeply stained. Interestingly, as the depth of root penetration increased, the staining became more pronounced (**Figures 4G–K**), consistent with early *TaHAK13* expression in roots and root tips at the seedling stage. Epidermal hairs (**Figure 4N**) and flowers (**Figure 4P**) were only stained lightly. Overall, the *TaHAK13* promoter predominantly drove GUS expression in mature roots and the vascular tissues of transgenic *Arabidopsis* plants, and the level of root-specific expression was influenced by developmental stage.

### 3.4 Functional complementation of *TaHAK13* in the yeast strain CY162

**Figure 5:**
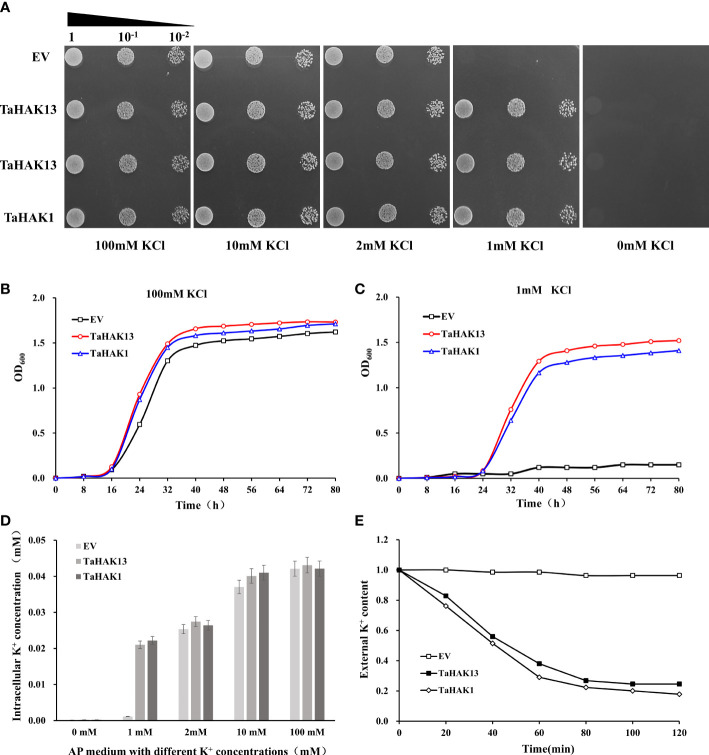
*TaHAK13* complementarity analysis in a Trk1 and Trk2 K^+^ uptake system deficient yeast strain **(A)** Growth of mutant CY162 in AP-U solid media with different concentrations of K^+^. CY162 was transformed with the empty vector p416 (EV), *TaHAK13*-p416 (TaHAK13), or *TaHAK1*-p416 (TaHAK1). After serial dilution, each strain was added to an agar plate for culture. **(B)** Growth curves for CY162 transformed with the empty vector, *TaHAK13*, or *TaHAK1* in AP-U liquid medium supplemented with 1mM K^+^ or 100 mM K^+^. **(C)** Determination of the K^+^ content in CY162 transformed with the empty vector p416, *TaHAK13*, or *TaHAK1* in solid AP-U media with various concentrations of K^+^. **(D)** K^+^ depletion experiment in the presence of 1 mM K^+^ in AP-U medium. CY162 (transformed with p416, *TaHAK13*, or *TaHAK1*) was subjected to K^+^ starvation for 4 h before beginning the experiment. The K^+^ content in the buffer was measured at intervals of 2 h.

The budding yeast *S. cerevisiae* has been shown to be an excellent model for studying ion transport and ion homeostasis (Mao et al., 2022). Mutant strains lacking their own ion transport systems serve as an efficient tool for the molecular study of higher eukaryote transporters *via* their expression in yeast cells (Xu et al., 2008). *TaHAK13* was inserted into the yeast strain CY162 to explore the K^+^ sensitivity of the TaHAK13 transporter. CY162 is defective in high-affinity potassium uptake and cannot grow on low K^+^ (≤1 mM) AP plates. A yeast complementation experiment was conducted on solid AP-U media with different K^+^ levels (0, 1, 2, 10, or 100 mM KCl). The p416-*TaHAK1* recombinant plasmid was transferred into CY162 for use as a positive control, while the p416 vector was transferred into CY162 to create a negative control. All test strains (including those with transgenes or the empty vector) grew uniformly on AP plates with 2, 10, or 100 mM KCl (**Figure 5A**). With only 1 mM KCl, the yeast strain transformed with *TaHAK13* showed similar growth to the positive control (*TaHAK1*); growth was poor for the empty vector transformant (negative control) (**Figure 5A**). Therefore, *TaHAK13* can restore growth in CY162 on low K^+^ media and has K^+^ transport ability.

As the RT-qPCR results revealed that *TaHAK13* can respond to salt stress, both the empty vector p416 and *TaHAK13-*p416 were transferred into the yeast strain AXT3K respectively. AXT3K does not possess any of the major endogenous sodium transporters essential for salt tolerance and is incapable of growing on AP plates with NaCl concentrations greater than 10 mM. Yeast drop experiments on AP media with different salt concentrations (0, 10, 20, 30, and 50 mM NaCl) were carried out. The growth of AXT3K transformed with *TaHAK13*-p416 and p416 was similar and neither survived at higher NaCl concentrations (**Figure S2**), indicating that TaHAK13 does not transport Na^+^.

### 3.5 Yeast growth curve and determination of intracellular and extracellular K^+^ in the yeast strain CY162

Patterns of yeast cell growth in AP-U liquid media with different concentrations of K^+^ further confirmed the growth restoration ability of the *TaHAK13* transformant. At 100 mM KCl, the transgenic yeast strain CY162 containing *TaHAK13* had the same growth rate as that of the transgenic yeast strain *TaHAK1* (positive control) and empty vector (negative control). At 1 mM KCl, *TaHAK13* and *TaHAK1* transformants grew normally, but the yeast strain transformed with an empty vector exhibited little growth (**Figure 5B**).

To further characterize the relationship between CY162 growth and K^+^ absorption, the K^+^ content of yeast strains cultured with various K^+^ concentrations was measured. Yeast expressing *TaHAK13*, *TaHAK1* (positive control), or an empty vector (negative control) maintained a stable K^+^ intracellular concentration when 100, 10, or 2 mM of external K^+^ was added to the AP liquid medium. However, under K^+^ stress (1 mM K^+^ AP medium), only yeast expressing *TaHAK13* or *TaHAK1* showed effective K^+^ absorption; the empty vector transformant showed growth deficits as the K^+^ content declined (**Figure 5C**). Meanwhile, CY162 transformed with *TaHAK13* or *TaHAK1* depleted the available K^+^ in the culture medium (1 mM), but no such depletion was observed by the empty vector strain (**Figure 5D**). In conclusion, *TaHAK13* was regulated by intracellular K^+^ and strictly controlled the intracellular K^+^ content to maintain ion balance.

### 3.6 Functional verification of *TaHAK13* in *Arabidopsis thaliana*

**Figure 6:**
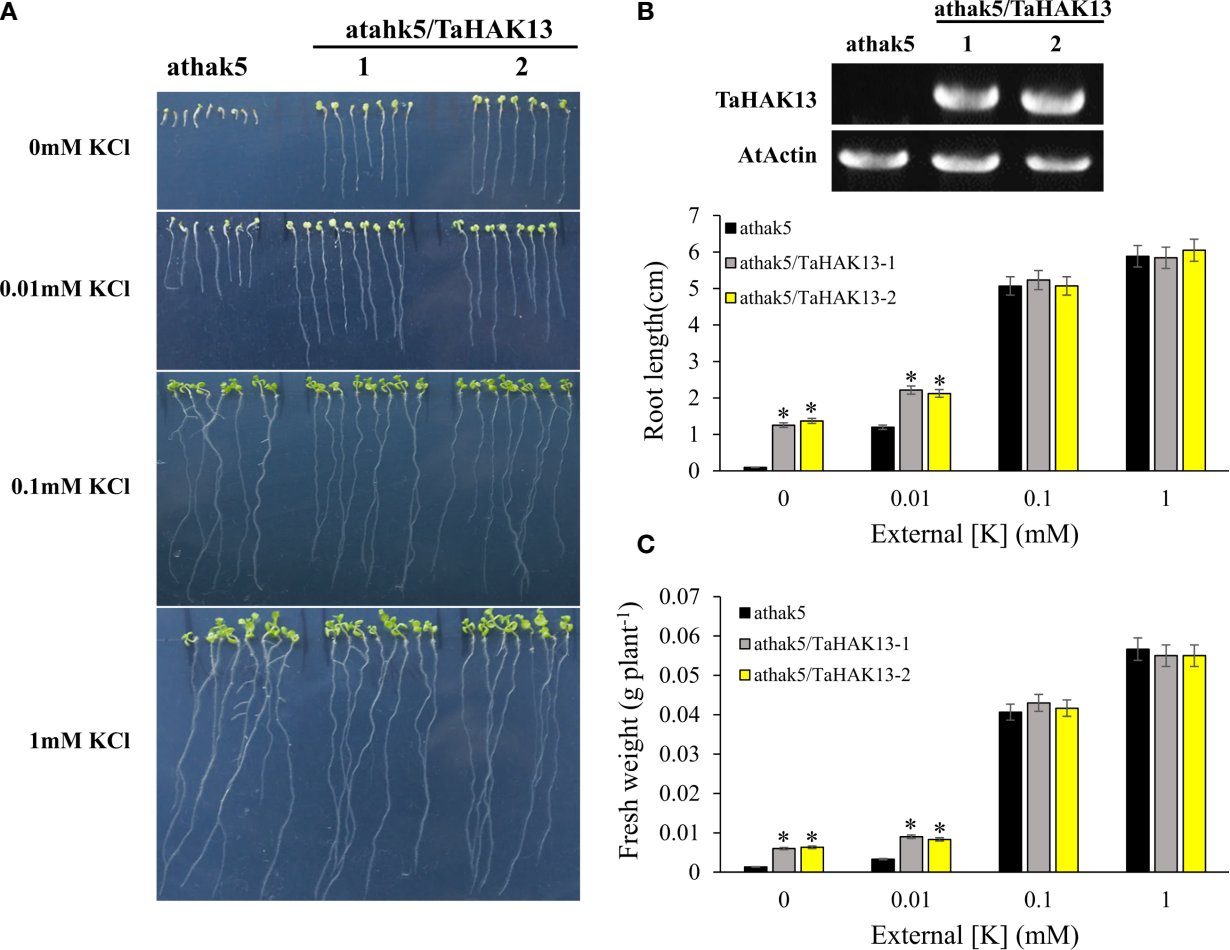
The expression of *TaHAK13* rescued the sensitive phenotype of *Arabidopsis* mutant *athak5* under low potassium condition. **(A)** The *athak5* mutant and *TaHAK13* transgenic lines (*athak5/TaHAK13*) were grown on MS media with different K^+^ concentrations for 10 d. **(B)** The expression of *TaHAK13* in *athak5* and the transgenic lines (*athak5/TaHAK13*) was analyzed using RT-PCR. **(C)** The root length in each bar represents the average root length of 20 seedlings from three independent experiments. **(D)** The plant fresh weight in each bar represents the average fresh weight of 20 seedlings from three independent experiments. The Student t-test (*p<0.05) was used to analyze the statistical significance.

**Figure 7:**
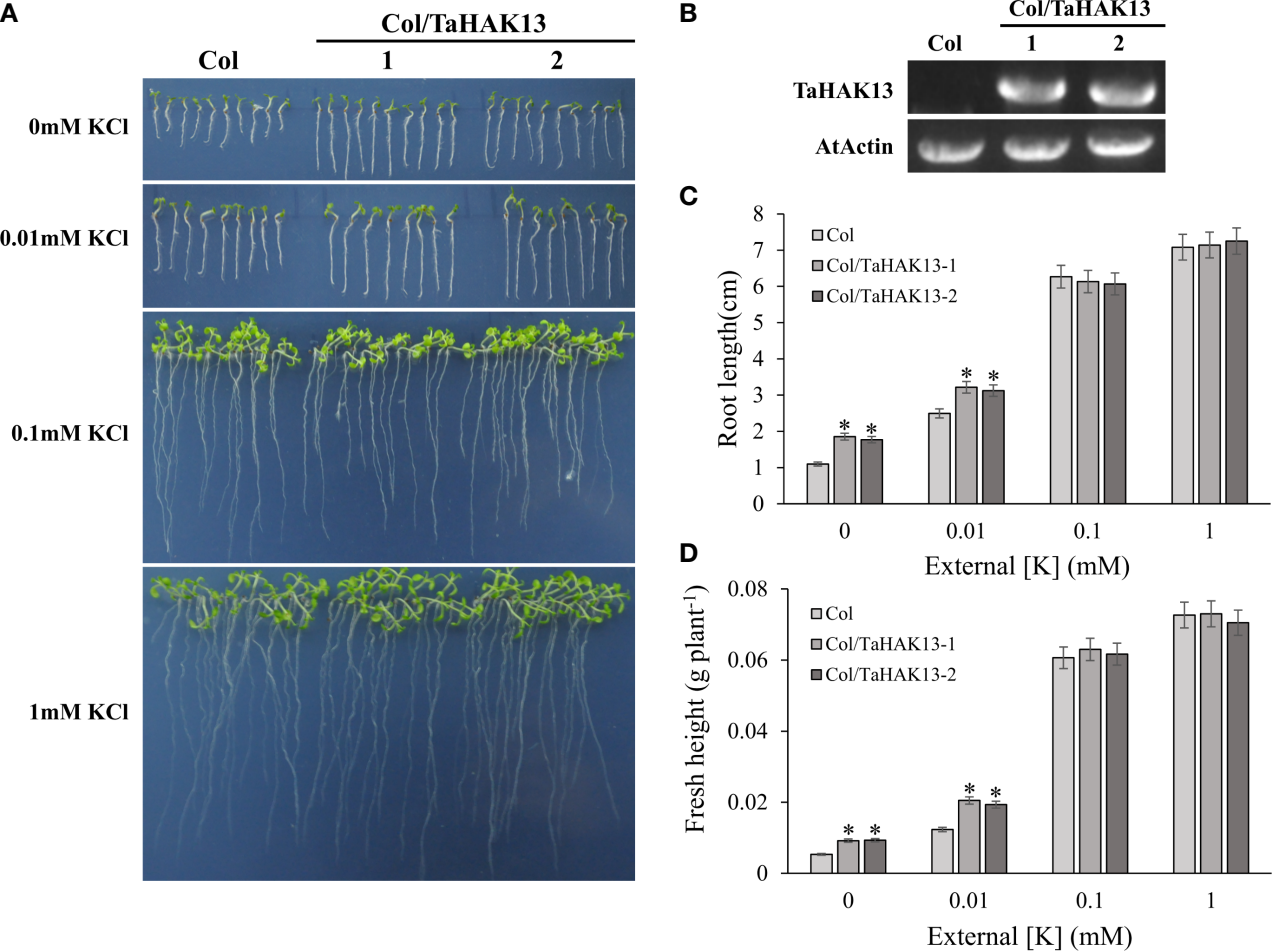
Functional verification of *TaHAK13* in wild-type *Arabidopsis thaliana.* **(A)** Wild-type (Col) and transgenic lines (Col/*TaHAK13*) were grown on MS media with different K^+^ concentrations for 10 d. **(B)** The expression of *TaHAK13* in Col and transgenic *Arabidopsis thaliana* was quantified *via* RT-PCR. **(C)** The root length of the plant in each bar represents the average root length of 20 seedlings from three independent experiments. **(D)** The fresh weight of plants in each bar represents the average fresh weight of 20 seedlings from three independent experiments. The Student t-test (*p<0.05) was used to analyze the statistical significance.

The *Arabidopsis thaliana* mutant *athak5* is sensitive to low potassium, and its primary root length is shorter than that of wild-type plants (Gierth et al., 2005; Pyo et al., 2010). To further characterize the function of *TaHAK13* in plants, it was amplified and expressed in the *Arabidopsis* mutant *athak5* and the wild-type (Col). Under normal potassium conditions (0.1 and 1 mM KCl), the root length and fresh weight of the complementary lines (*athak5/TaHAK13*) were not significantly different from those of the mutant lines (*athak5*), but when growing under low K conditions (0 and 0.01 mM KCl), the *athak5* mutant showed serious growth defects. The expression of *TaHAK13* rescued the sensitive phenotype of *athak5* (**Figures 6A, B**, **S5**), and the root length and fresh weight of the two transgenic lines were significantly higher than those of the *athak5* mutant (**Figures 6C, D**). Similar patterns were seen for the over-expression lines (Col/*TaHAK13*) and wild-type lines (Col) (**Figures 7A, B**). Under normal potassium conditions (0.1 and 1 mM KCl), the root length and fresh weight of Col/*TaHAK13* transgenic lines did not differ from those of the Col non-transgenic lines, while under low K conditions (0 and 0.01 mM KCl), the expression of *TaHAK13* increased plant tolerance of low potassium (**Figures 7C, D**). These results further confirm that *TaHAK13* acts as a high affinity potassium transporter that mediates K^+^ uptake in plants under low potassium conditions.

### 3.7 Effect of *TaHAK13* expression on potassium uptake by *Arabidopsis* roots

**Figure 8:**
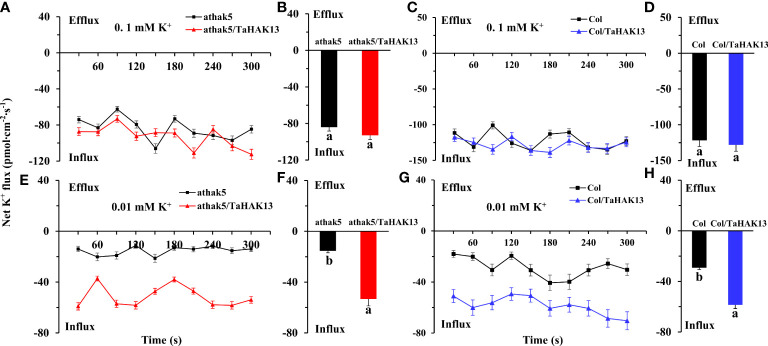
Effect of *TaHAK13* expression on the net K influx in primary root meristem supplied with different concentrations of (K) **(A, C)** The net K influx of *Arabidopsis thaliana* mutant *athak5* and its complementary line (*athak5*/*TaHAK13*) over 8 min (see ‘Materials and methods’). **(B, D)** Mean net K influx of *athak5* and *athak5*/*TaHAK13* when 0.1 mM K^+^ **(A)** or 0.01 mM K^+^ **(C)** was supplied; average values were taken over the whole eight minutes of influx data. **(E, G)** The net K influx of wild-type *Arabidopsis thaliana* (Col) and its overexpression line (Col/*TaHAK13*) over 8 min. **(F, H)** Mean net K influx of Col and Col/*TaHAK13* when 0.1 mM K^+^ **(A)** or 0.01 mM K^+^ **(C)** was supplied; average values were taken over the whole eight minutes of influx data. Significant differences between transgenic lines and their respective non-transgenic lines are indicated by different letters.

To determine whether *TaHAK13* is needed for potassium acquisition in *Arabidopsis* roots in low potassium environments, the net K influx in seedling primary roots was measured using non-invasive micro-test technology (NMT) (**Figure S6**). The net K influx was then compared between the *TaHAK13* complementary lines (*athak5/TaHAK13*) and overexpression lines (Col/*TaHAK13*) and their respective wild-types. In eight minutes of measurement, no differences were detected between the *athak5/TaHAK13* lines and the *athak5* mutant when seedlings were supplied with 0.1 mM K^+^ over the measurement period (**Figures 8A, B**). However, when the K^+^ concentration supplied was 0.01 mM, the net K influx was larger in *athak5/TaHAK13* lines versus the *athak5* mutant over the six minutes measurement (**Figure 8C**). On average, *TaHAK13* expression in the *athak5* mutant increased the net K influx about 3.5 times (**Figure 8D**). A similar pattern was seen for the Col/*TaHAK13* lines versus wild-type. When 0.01 mM K^+^ was supplied, the wild-type had a much lower net K influx, only about 50% of that observed for the Col/*TaHAK13* lines (**Figures 8G, H**). Increasing the K^+^ concentration from 0.01 to 0.1 mM dramatically augmented the net K influx in both Col/*TaHAK13* lines and wild-type; over time, the K influx in the wild-type largely caught up, finishing only about 5% less than the Col/*TaHAK13* influx rate (**Figures 8E, F**). Therefore, *TaHAK13* is directly involved in the acquisition of root K^+^, especially in low potassium conditions.

### 3.8 TaHAK13 interacted with TaNPF5.10 and TaNPF6.3

**Figure 9:**
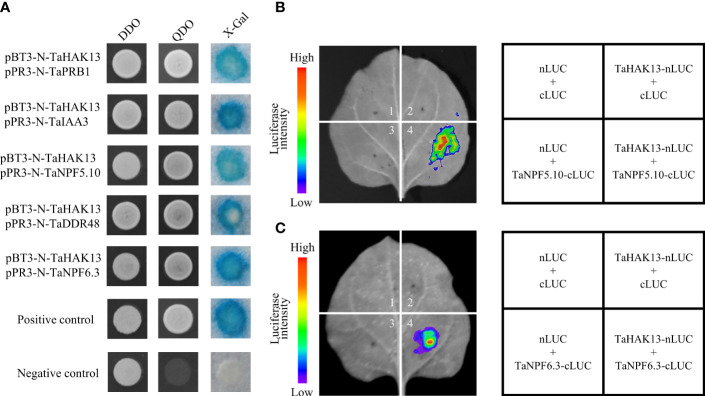
The interaction of TaHAK13 with TaNPF5.10 and TaNPF6.3. **(A)** The interaction of TaHAK13 with screened proteins was verified using a split-ubiquitin yeast two-hybrid system; different protein combinations were detected on appropriate screening media (SD/-L-T, SD/-A-/H/-L/-T, SD/-A/-H/-L/-T+20μg/mL X-Gal). **(B)** LCA of the interaction between TaHAK13 and TaNPF5.10. **(C)** LCA of TaHAK13 and TaNPF6.3. As shown in the graphics, various combinations were used to co-transform tobacco leaves. Luciferase signals were captured by a NightSHADE LB 985 plant *in vivo* imaging system.

The full-length *TaHAK13* sequence was inserted into a pBT3-N vector for use as a bait vector to screen for protein interactions in wheat. Self-activation and toxicity tests revealed that the bait vector was normally expressed in a yeast system, was non-toxic to yeast, and had no self-activation, so it was used to subsequent screening experiments (**Figure S7**). After screening, five genes were identified; these genes were involved in many aspects of plant disease resistance, including signal transduction, stress resistance, and nutritional stress resistance (**Table S4**). The above five cDNA sequences were inserted into a prey vector (pPR3-N); the bait and prey vectors were then transformed into the yeast strain NMY51. NMY51 was grown on SD/-Leu/-Trp (DDO) and SD/-His/-Leu/-Trp/-Ade (QDO) medium for 3 d, and then X-Gal was used for identification. Except for the negative control, the positive control and the verified transformation solution both grew normally and turned blue on the QDO medium (**Figure 9A**). Interestingly, the genes *TaNPF5.10* and *TaNPF6.3* belong to the NPT/PTR family of wheat, which plays an important role in the absorption, transport, and distribution of nitrate in plant cells, tissues, and organs. Therefore, a luciferase complementation assay (LCA) was carried out to assess protein interactions. Only the co-transformed areas of TaHAK13 and TaNPF5.10 or TaNPF6.3 emitted fluorescence. The transformed empty vector (nLUC + cLUC) and other combinations (TaHAK13-nLUC + cLUC, nLUC + TaNPF5.10-cLUC, and nLUC + TaNPF6.3-cLUC) did not emit fluorescence (**Figures 9B, C**), suggesting that TaHAK13 interacts with both TaNPF5.10 and TaNPF6.3.

## 4 Discussion

Soil K^+^ concentrations are often low for plant growth and development, meaning that plants often experience low potassium stress (Wang and Wu, 2009). The KUP/HAK/KT family of potassium transporters constitutes the primary system for K^+^ uptake in plants under low-K^+^ concentrations. Many high affinity potassium transporter genes have been identified to date, such as *AtHAK5* (*Arabidopsis thaliana*), *GhHAK5* (*Gossypium hirsutum*), *HvHAK1* (*Hordeum vulgare*), *LeHAK5* (*Lycopersicon esculentum*), and *OsHAK1* (*Oryza sativa*) (Santa-María et al., 1997; Bañuelos et al., 2002; Wang et al., 2002; Gierth et al., 2005; Chao et al., 2018). Studying these genes can provide a guide to the molecular mechanisms underlying K^+^ transport. In this study, a wheat gene that is homologous to *AetHAK13* in *Aegilops* was identified and researched (**Figures S3**, **S4**).

### 4.1 *TaHAK13* expression occurs mainly in roots and is influenced by tpdevelopmental stage

To explore how *TaHAK13* functions in plants, the *TaHAK13* promoter was cloned from the hexaploid common wheat variety Yunong 804. The promoter sequence was found to contain a TATA-box and CAAT-box, two key components responsible for initiating and regulating transcription in eukaryotes (Luo et al., 2015). The *TaHAK13* promoter also contained many photo-responsive elements, such as an ACE, G-box, GT1-motif, and others. Both the G-box (Heng et al., 2019) and GT1-motif (Zhao et al., 2012) are essential for genes to respond to light signals. In addition, the *TaHAK13* promoter also contained a MYB binding site and a WRKY-binding W-box, so *TaHAK13* expression is likely also regulated by upstream transcription factors (**Table S3**). Previous research has shown that HAK family genes, such as *OsHAK21* (Shen et al., 2015), *AtKUP7* (Han et al., 2016), *SiHAK10* (Liu et al., 2019), and *SiHAK20* (Wang et al., 2020), are mainly expressed in roots. The tissue-specificity of *TaHAK13* was examined, and expression was found to be mainly focused in plant veins, stems, and root tips at the seedling stage; in mature plants *TaHAK13* was highly expressed in the roots, suggesting that expression varied with developmental stage. Expression was higher in mature plants versus seedlings, consistent with the characteristics of most high affinity potassium transporters (**Figure 4**). Most HAK family genes belonging to cluster I are induced by low potassium stress, such as *OsHAK5* (Yang et al., 2014), *AtHAK5* (Nieves-Cordones et al., 2019), and *OsHAK16* (Feng et al., 2019). GUS staining was stronger in low potassium conditions versus normal conditions, indicating higher gene expression level. In addition, *OsHAK8* (part of cluster II) is also induced by low potassium stress (Wang et al., 2021). Therefore, the expression of *TaHAK13* in plants seems to be affected by low potassium stress.

### 4.2 *TaHAK13* mediates K^+^ absorption and maintains K^+^ homeostasis

In rice, *OsHAK1* improves the growth of yeast at KCl concentrations of 0.05-1 mM. Furthermore, for any K concentration, yeast expressing *OsHAK1* is also more tolerant to salt stress (Chen et al., 2015). Compared to yeast transformed with an empty pYES2 vector, yeast strains expressing *OsHAK16* can tolerate up to 200 mM NaCl (Feng et al., 2019). In addition, *OsHAK16* expression improves the growth of the low potassium-sensitive yeast mutant R5421 at 0.1, 1, and 10 mM K^+^ supply rates. Collectively, these results suggest that *OsHAK16* can enhance K^+^ absorption in yeast cells (Feng et al., 2019). In this study, *TaHAK13*, *TaHAK1*, and an empty vector were inserted into the yeast strain CY162. The CY162 strain transformed with an empty vector (p416) did not grow normally on low potassium medium. However, the addition of *TaHAK1* or *TaHAK13* restored the defective phenotype of CY162; these strains grew normally on AP medium with 1 mM KCl, suggesting that *TaHAK13* plays an important function in K^+^ absorption (**Figure 5**). In previous studies, WΔ3 yeast cells expressing *CaHAK1* can survive on the media with K^+^ concentrations of less than 1 μM, but this ability is inhibited by micromolar concentrations of $\text{N}{\text{H}}_{4}^{+}$ (Martínez-Cordero et al., 2004). Under low potassium stress, yeast strains transformed with *SiHAK1* show higher growth rates than positive controls (Zhang et al., 2018). In addition, the expression of *HbHAK1* in yeast strain CY162 promotes K^+^ absorption when potassium levels are extremely low, and reduces sodium toxicity to support yeast cell survival under high salt stress (Zhang et al., 2020). Here, *TaHAK13*, *TaSOS1*, and an empty vector were transferred into the mutant AXT3K, which has high salt sensitivity. The *TaHAK13* mutant exhibited poor growth under high sodium conditions, as did that with an empty vector; only the positive control (*TaSOS1*) grew well. Therefore, TaHAK13 does not transport Na^+^. Presumably, the main function of TaHAK13 is as a K^+^ transporter that shows Na^+^ sensitivity (**Figure S2**).

*Arabidopsis thaliana* mutants (*athak5)* showed serious growth defects when grown on MS medium without additional K^+^. The expression of short awn barley *HbHAK1*, millet *SiHAK1*, corn *ZmHAK5*, or rice *OsHAK21* restored growth to similar levels as the wild-type under low potassium conditions (Shen et al., 2015; Zhang et al., 2018; Qin et al., 2019., Zhang et al., 2020). Loss of function of *KUP7* and *KUP9* in *Arabidopsis thaliana* results in short roots and yellow leaves on low potassium medium; the K^+^ absorption rate and potassium content in xylem sap also decreases (Han et al., 2016; Zhang et al., 2020). To assess whether *TaHAK13* has a similar function in plants, *TaHAK13* was introduced into the mutant *athak5*. Under low potassium conditions (0 mM KCl and 0.01 mM KCl), *athak5* had short roots and yellow leaves, while the *TaHAK13* transgenic strain did not show similar deficits: root length and fresh weight were significantly higher than in *athak5* (**Figure 6** and **S5**). These results indicate that TaHAK13 mediated the absorption and transportation of K^+^. In addition, the expression of some HAK/KUP/KT family genes can enhance plant salt tolerance. For example, *oshak16* knockout lines show reduced K^+^ absorption and a lower K^+^/Na^+^ ratio, while *OsHAK16* overexpression lines have higher K^+^ absorption and greater root-to-shoot transport, thus improving salt tolerance (Feng et al., 2019). Similarly, wild-type and *zmhak4* knockout mutants do not differ under normal conditions, but under salt stress (100 mM NaCl), *zmhak4* knockout mutants are about 15% smaller than wild-type controls, suggesting that *ZmHAK4* promotes salt tolerance by maintaining a steady state (of Na^+^ and K^+^) and constant K^+^/Na^+^ ratio (Zhang et al., 2019). Here, the expression of *TaHAK13* in *Arabidopsis thaliana* may improve the salt tolerance of plants.

### 4.3 TaHAK13 interacts with TaNPF5.10 and TaNPF6.3 to influence the cell membrane

The DUAL membrane system is a yeast two-hybrid system of membrane proteins mediated by split-ubiquitin. It provides a method of *in vivo* protein analysis different from the conventional yeast two-hybrid system, which makes it possible to analyze interactions among membrane proteins (Johnsson and Varshavsky, 1994). Here, *TaHAK13* was inserted into a pBT3-N bait vector, and a wheat cDNA library was screened for proteins interacting with the protein encoded by this gene. Among the proteins identified, TaNPF5.10 and TaNPF6.3 had the strongest interactions with TaHAK13 (**Figure 9**), so we speculate that this interaction may co-regulate the absorption of K^+^ and $\text{N}{\text{O}}_{3}^{-}$ by plants. In *Arabidopsis thaliana*, the *nrt1.1* knockout mutant showed poor K^+^ absorption and root-shoot distribution, as well as growth stagnation when K^+^ is restricted. These K^+^ absorption-related interactions depend on H^+^ consumption mechanisms related to NRT1.1-mediated H^+^/ $\text{N}{\text{O}}_{3}^{-}$ co-metabolism (Fang et al., 2020). The interactions between K^+^ and different N forms is realized by NRT1.5 modulation of root-derived ethylene signals that regulate K^+^ transport from root to shoot. $\text{N}{\text{H}}_{4}^{+}$ upregulates the transcription activity of ET-insensitive 3 (EIN3) but inhibits the expression of *NRT1.5*. The addition of $\text{N}{\text{O}}_{3}^{-}$ strongly inhibits the activity of EIN3, while upregulating the expression of *AtNRT1.5* and increasing the K^+^ concentration (Chen et al., 2021). Plants can sense the ratio of K^+^/ $\text{N}{\text{O}}_{3}^{-}$ in the soil, adjusting the K^+^/ $\text{N}{\text{O}}_{3}^{-}$ transport ratio between roots and shoots to maintain a balance of these ions in their tissues. Transcription factor *MYB59* aids in this process by regulating the transcription of *AtNRT1.5*/*AtNPF7.3* in response to low potassium stress (Du et al., 2019). Studies have reported positive interactions between potassium and nitrogen in wheat, i.e., high potassium can alleviate ammonium nitrogen stress, improving growth by promoting the absorption of nutrients and the production of assimilation products (Guo et al., 2019). In this study, two genes were identified interacting with *TaHAK13* using a MbY2H and a luciferase complementation assay (LCA). *TaNPF5.10* and *TaNPF6.3* belong to the NPT/PTR family in wheat; this family plays an important role in the absorption, transport, and distribution of nitrate nitrogen in plant cells, tissues, and organs. In addition, all three proteins were localized in the cell membrane and had a transmembrane domain (**Figures S8**, **S9**); therefore, TaHAK13 most likely interacts with TaNPF5.10 and TaNPF6.3 within the plasma membrane.

TaHAK13 is a member of cluster II that is widely distributed in plant tissues, including in roots, stems, leaves, and flowers. The members of cluster II not only participate in K^+^ absorption, but also play a role in growth regulation. Isotope ^32^P labeling has been used to analyze the expression of *AtKUP1*, *AtKUP2*, *AtKUP3*, and *AtKUP4* in different tissues under normal potassium supply; all four genes were expressed in roots, leaves, and flowers, without tissue expression specificity (Kim et al., 1998). The HAK protein of cluster II plays a variety of physiological roles in plants, such as promoting K^+^ absorption, maintaining intracellular K^+^ concentrations, and participating in cell expansion and growth. It indirectly affects the transmembrane transport of root auxin by regulating the intracellular H^+^ content, thus initiating the development of root hairs and the geotropism of roots (Rodrίguez-Navarro, 2000). Therefore, TaHAK13 may also participate in K^+^ absorption and cell expansion and growth, but further research is required to verify this hypothesis.

## 5 Conclusions

In this study, the TaHAK13 gene was cloned from wheat and its function characterized. RT-qPCR showed that TaHAK13 expression was up-regulated under drought, low potassium, and salt stress. GUS staining indicated that TaHAK13 was mainly expressed in the leaf veins, stems, and root apex in *Arabidopsis thaliana*, and expression varied with developmental stage. The subcellular localization analysis illustrated that TaHAK13 was located to the cytoplasmic membrane. In yeast and *Arabidopsis*, the overexpression of TaHAK13 improved their ability to absorb K^+^ under low potassium condition, but did not have the ability to transport Na^+^. Membrane-based yeast two-hybrid (MbY2H) and luciferase complementation assays (LCA) showed that TaHAK13 interacted with TaNPF5.10 and TaNPF6.3 proteins. Overall, our study revealed the role of TaHAK13 in plants and the mechanism of low potassium tolerance in plants.

## Data availability statement

The datasets presented in this study can be found in online repositories. The names of the repository/repositories and accession number(s) can be found in the article/**Supplementary Material**.

## Author contributions

YR and HX conceived and designed the experiments. YR, XC, WD, YD, YZ, and BL performed the experiments. XC and TL analyzed the data. YR wrote the manuscript. HX revised the article. All authors read and approved the submitted version.

## Supplementary material

Supplementary Figure 1 Real-time quantitative PCR expression of *TaHAK13* in wheat roots under normal conditions.

Supplementary Figure 2 Salt sensitivity of transgenic *TaHAK13* yeast. The mutant AXT3K grew on AP-U solid media with different concentrations of NaCl. AXT3K strain was transformed into empty vector (EV) or *TaHAK13* and positive control *TaSOS1*. Each strain was diluted continuously and dropped on agar plate for culture.

Supplementary Figure 3 Amino acid sequence alignment of HAK13-like K^+^ transporters from different plant species. The amino acid sequences of nine K^+^ transporters were aligned using DNAMAN software. These transporters included *TaHAK13* from wheat, *AtKUP9*, *AtKUP10* and *AtKUP13* from *Arabidopsis*, *AetHAK13* from Aegilops, *HvHAK13* from barley, *OsHAK13* from rice, *PvHAK13* from switchgrass, *SbHAK13* from sorghum, *BdHAK13* from brachypodium distachyon, *PhaHAK13* from Panicum hallii, *SiHAK13* from millet, *SvHAK13* from Green bristlegrass.

Supplementary Figure 4 Phylogenetic analysis of HAK13-like K^+^ transporters. phylogenetic tree was constructed by MEGA5.0 software using the neighbor-joining method, and statistical support for the nodes was assessed by bootstrap analysis (1000 replicates).

Supplementary Figure 5 Growth condition of *athak5* mutant and *TaHAK13* transgenic lines under normal and low potassium conditions. **(A)** *athak5* mutant and *TaHAK13* transgenic lines grew on MS medium and low potassium media for ten days. **(B)** The plant survival rate of *athak5* mutant and *TaHAK13* transgenic lines on MS medium and low potassium media.

Supplementary Figure 6 Measurement chart of net K^+^ flux in *Arabidopsis* roots. On the left side, the root zone of *Arabidopsis*, and on the right side, the flow sensor (3-5 μm) is used to test the root zone of *Arabidopsis* (150 μm from the root tip), and each planet is measured for eight minutes.

Supplementary Figure 7 MbY2H screening using TaHAK13 as a bait. **(A)** Schematic diagram of MbY2H screening. The cDNA library developed using the total RNAs of low potassium stressed wheat roots was screened using TaHAK13-pBT3-N as bait. **(B)** Self-activation and toxicity tests of bait vector TaHAK13-pBT3-N. **(C)** PCR analysis of positive clones. PCR amplification was performed for the positive clones using the primers pPR3-N-F and pPR3-N-R (**Table S2**).

Supplementary Figure 8 Predicted transmembrane domains of TaNPF5.10 and TaNPF6.3.

Supplementary Figure 9 Subcellular localization of TaNPF5.10 and TaNPF6.3 in tobacco leaves. 35S-GFP acted as the control.

Supplementary Table 1 Improved Hogland nutrient solution. Note: (1) Low potassium stress: the final concentration of K^+^ was 1 × 10^-5^mol·L^-1^, and K_2_SO_4_ was used to add K^+^. (2) Salt stress: NaCl with final concentration of 200 mM was added to the above nutrient solution. (3) Drought stress (20% PEG6000): PEG6000 with a final concentration of 20% was added to the above nutrient solution.

Supplementary Table 2 Primer sequences used in this study.

Supplementary Table 3 Analysis of cis-elements in the promoters of *TaHAK13*.

Supplementary Table 4 Functional analysis of TaHAK13 interacting protein.
