# Supplementary figures and images for: Wheat potassium transporter TaHAK13 mediates K+ absorption and maintains potassium homeostasis under low potassium stress
Source: Front Plant Sci. 2022 Dec 23;13:1103235. doi: 10.3389/fpls.2022.1103235 (PMC9816385; doi:10.3389/fpls.2022.1103235)

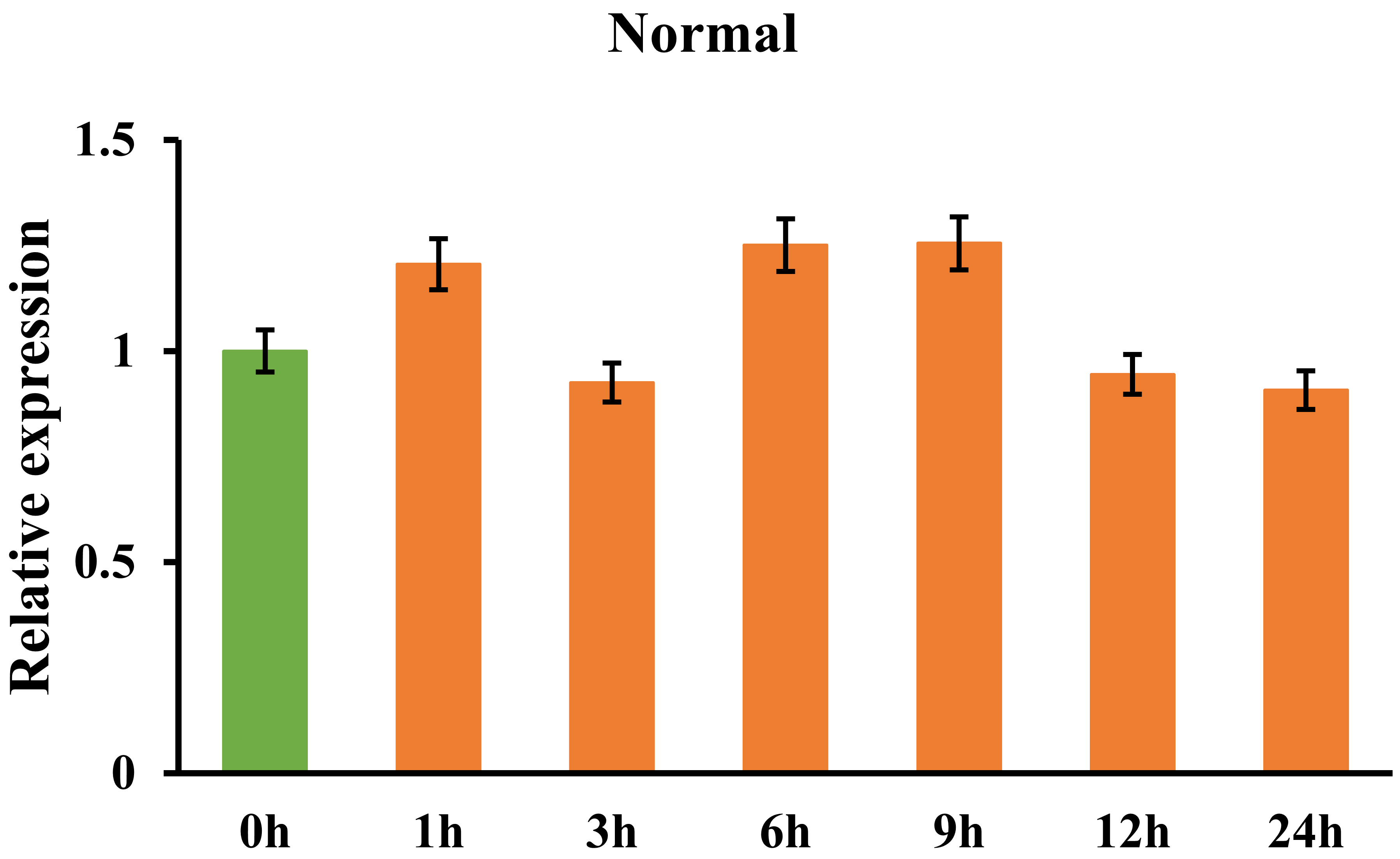

Supplement: Supplementary Figure 1 — Real-time quantitative PCR expression of TaHAK13 in wheat roots under normal conditions. [file Image_1.tif]

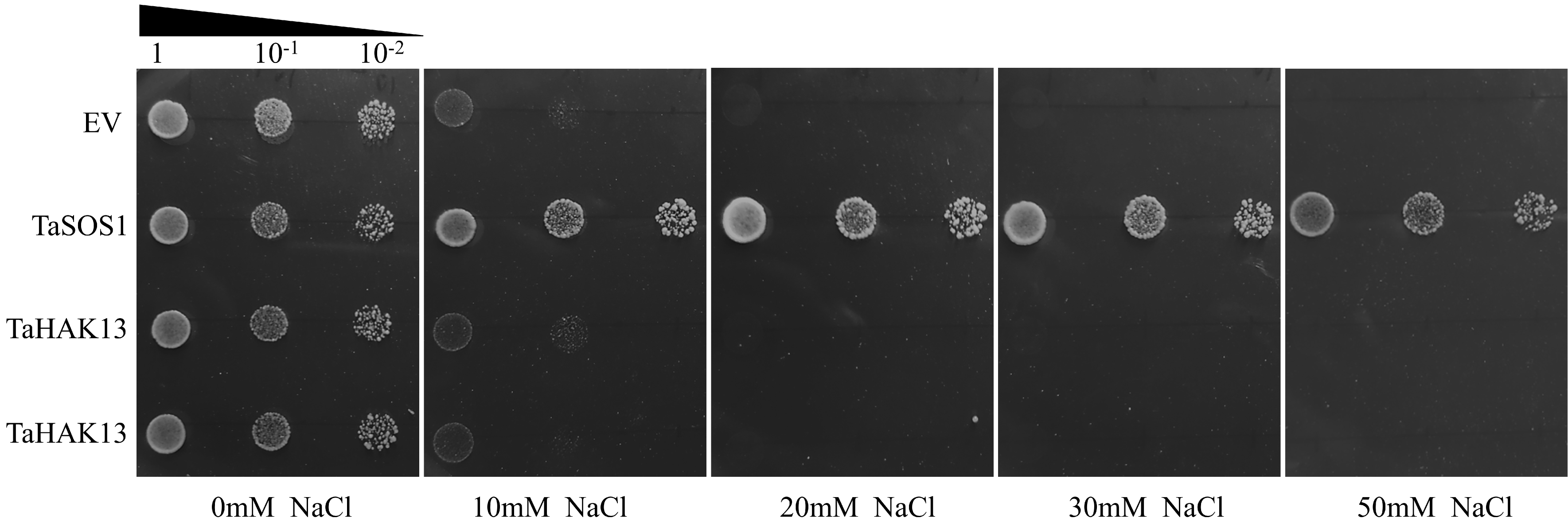

Supplement: Supplementary Figure 2 — Salt sensitivity of transgenic TaHAK13 yeast. The mutant AXT3K grew on AP-U solid media with different concentrations of NaCl. AXT3K strain was transformed into empty vector (EV) or TaHAK13 and positive control TaSOS1. Each strain was diluted continuously and dropped on agar plate for culture. [file Image_2.tif]

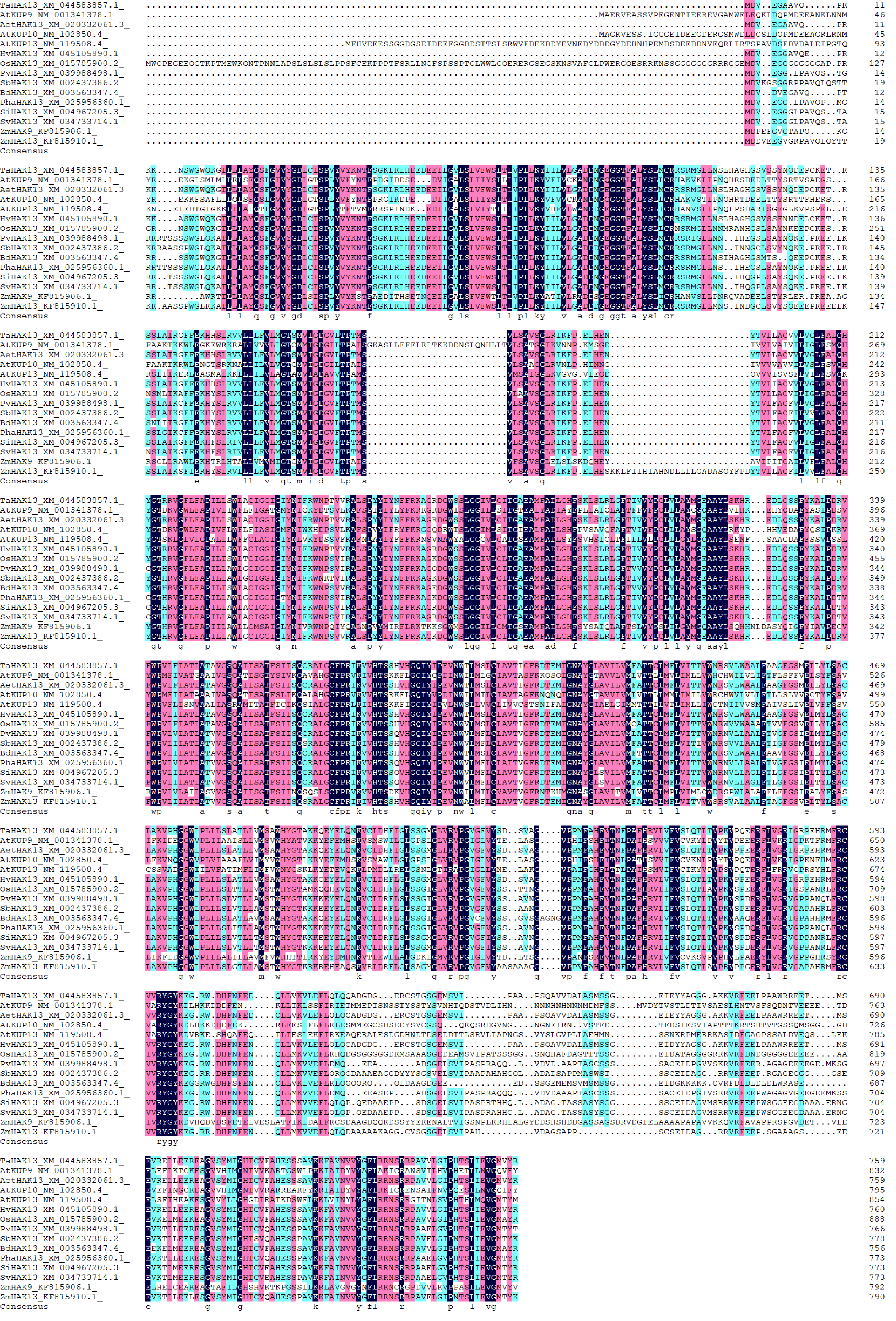

Supplement: Supplementary Figure 3 — Amino acid sequence alignment of HAK13-like K+ transporters from different plant species. The amino acid sequences of nine K+ transporters were aligned using DNAMAN software. These transporters included TaHAK13 from wheat, AtKUP9, AtKUP10 and AtKUP13 from Arabidopsis, AetHAK13 from Aegilops, HvHAK13 from barley, OsHAK13 from rice, PvHAK13 from switchgrass, SbHAK13 from sorghum, BdHAK13 from brachypodium distachyon, PhaHAK13 from Panicum hallii, SiHAK13 from millet, SvHAK13 from Green bristlegrass. [file Image_3.tif]

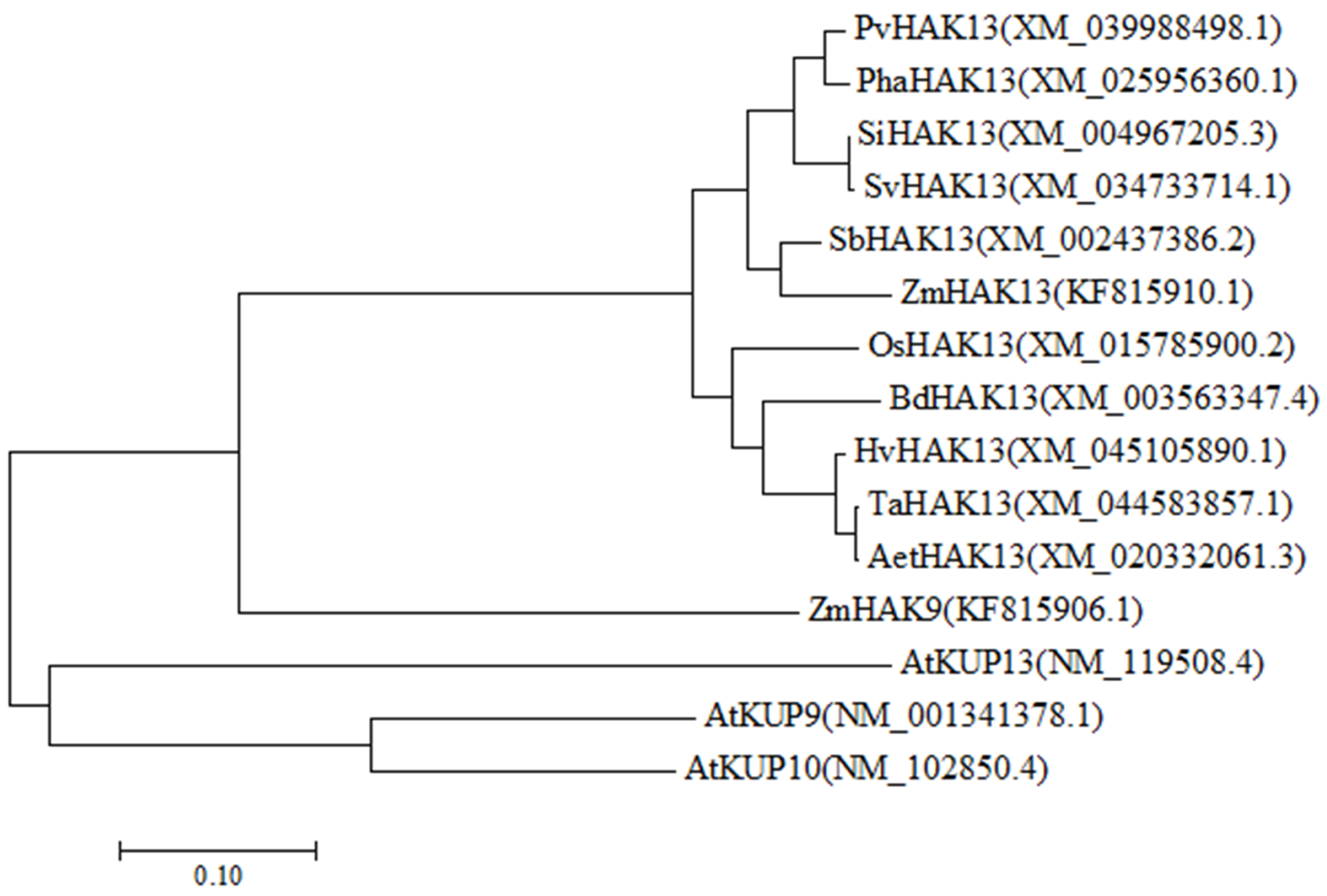

Supplement: Supplementary Figure 4 — Phylogenetic analysis of HAK13-like K+ transporters. phylogenetic tree was constructed by MEGA5.0 software using the neighbor-joining method, and statistical support for the nodes was assessed by bootstrap analysis (1000 replicates). [file Image_4.tif]

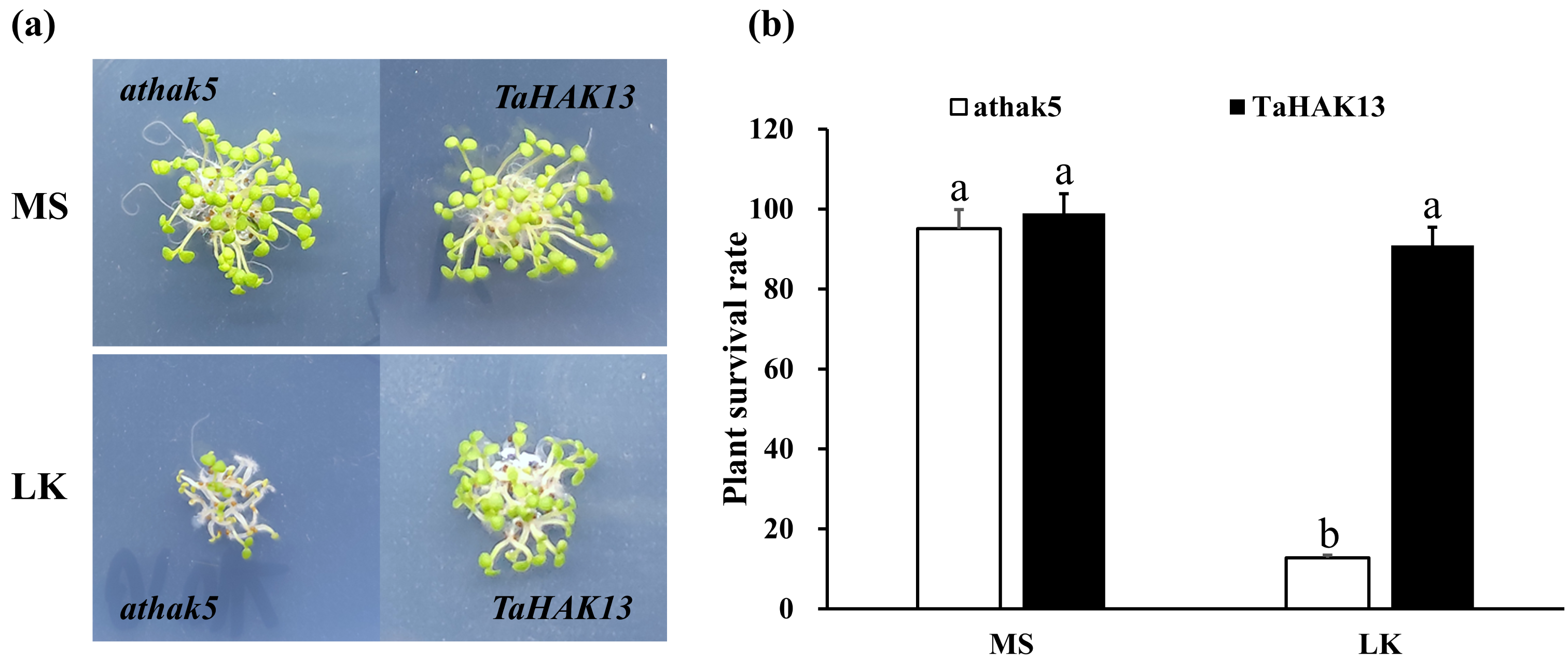

Supplement: Supplementary Figure 5 — Growth condition of athak5 mutant and TaHAK13 transgenic lines under normal and low potassium conditions. (A) athak5 mutant and TaHAK13 transgenic lines grew on MS medium and low potassium media for ten days. (B) The plant survival rate of athak5 mutant and TaHAK13 transgenic lines on MS medium and low potassium media. [file Image_5.tif]

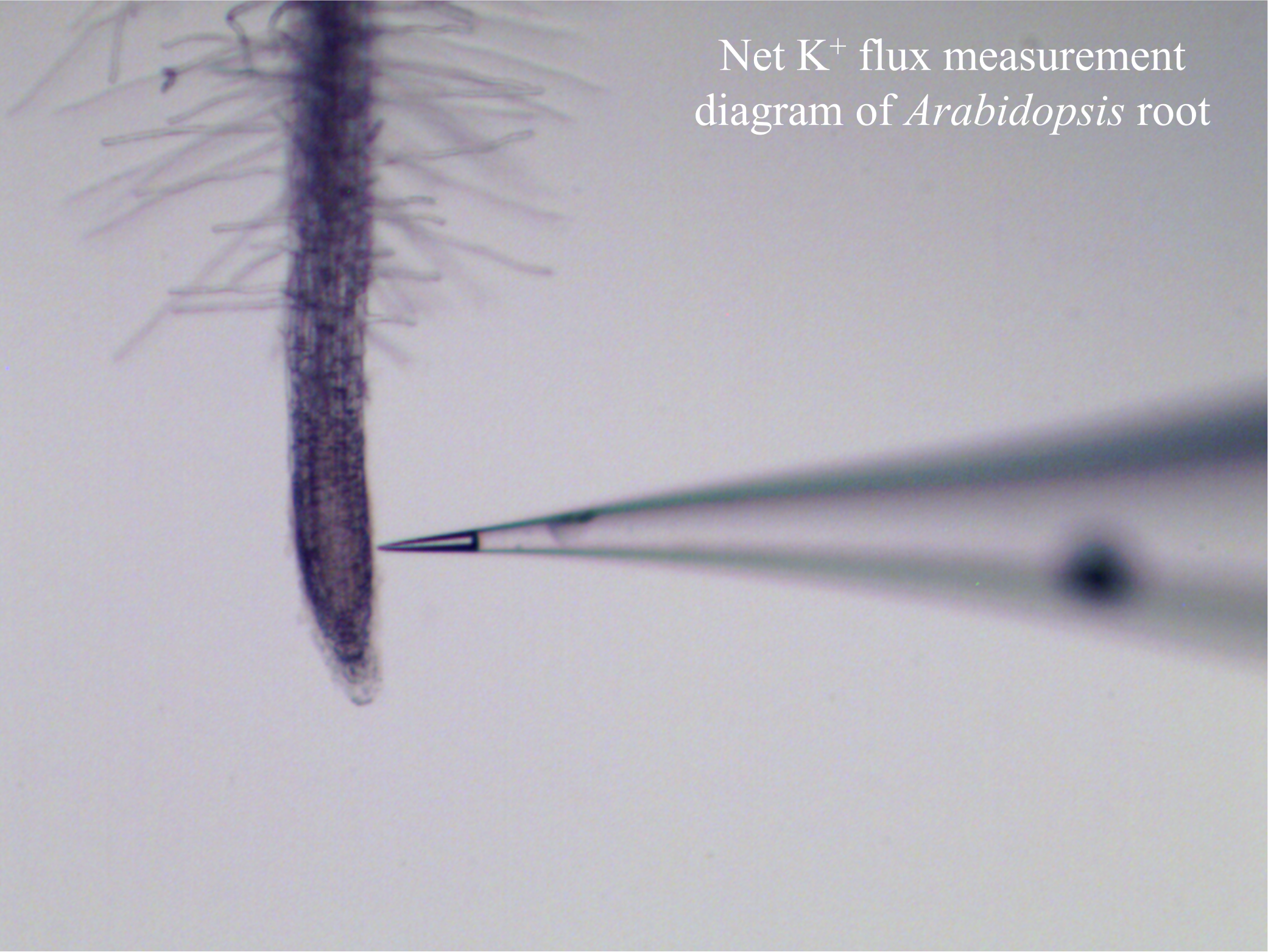

Supplement: Supplementary Figure 6 — Measurement chart of net K+ flux in Arabidopsis roots. On the left side, the root zone of Arabidopsis, and on the right side, the flow sensor (3-5 μm) is used to test the root zone of Arabidopsis (150 μm from the root tip), and each planet is measured for eight minutes. [file Image_6.tif]

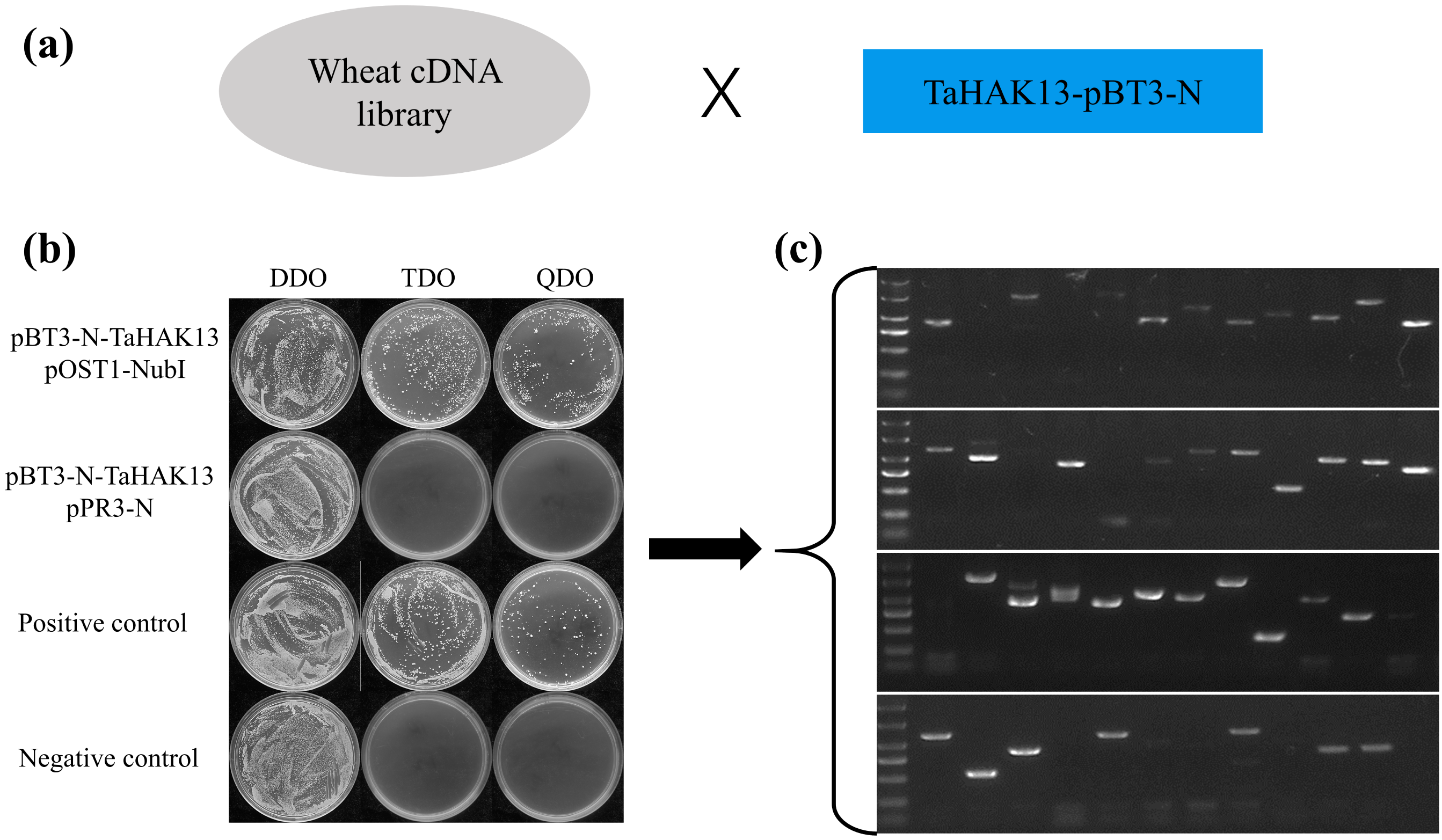

Supplement: Supplementary Figure 7 — MbY2H screening using TaHAK13 as a bait. (A) Schematic diagram of MbY2H screening. The cDNA library developed using the total RNAs of low potassium stressed wheat roots was screened using TaHAK13-pBT3-N as bait. (B) Self-activation and toxicity tests of bait vector TaHAK13-pBT3-N. (C) PCR analysis of positive clones. PCR amplification was performed for the positive clones using the primers pPR3-N-F and pPR3-N-R ( Table S2 ). [file Image_7.tif]

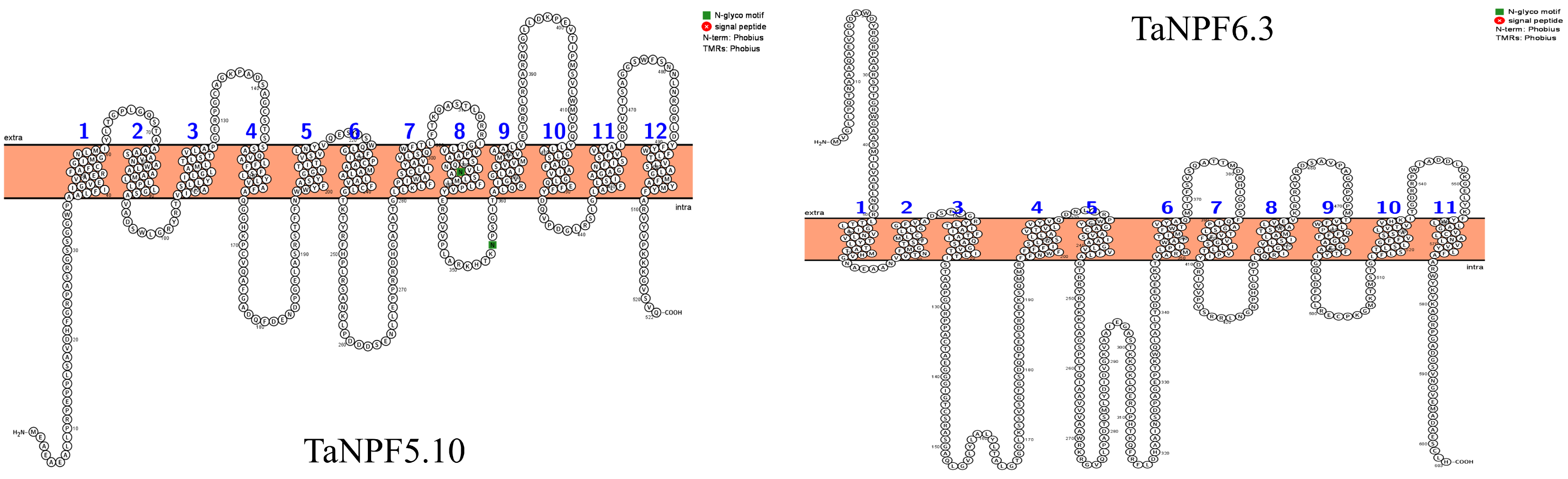

Supplement: Supplementary Figure 8 — Predicted transmembrane domains of TaNPF5.10 and TaNPF6.3. [file Image_8.tif]

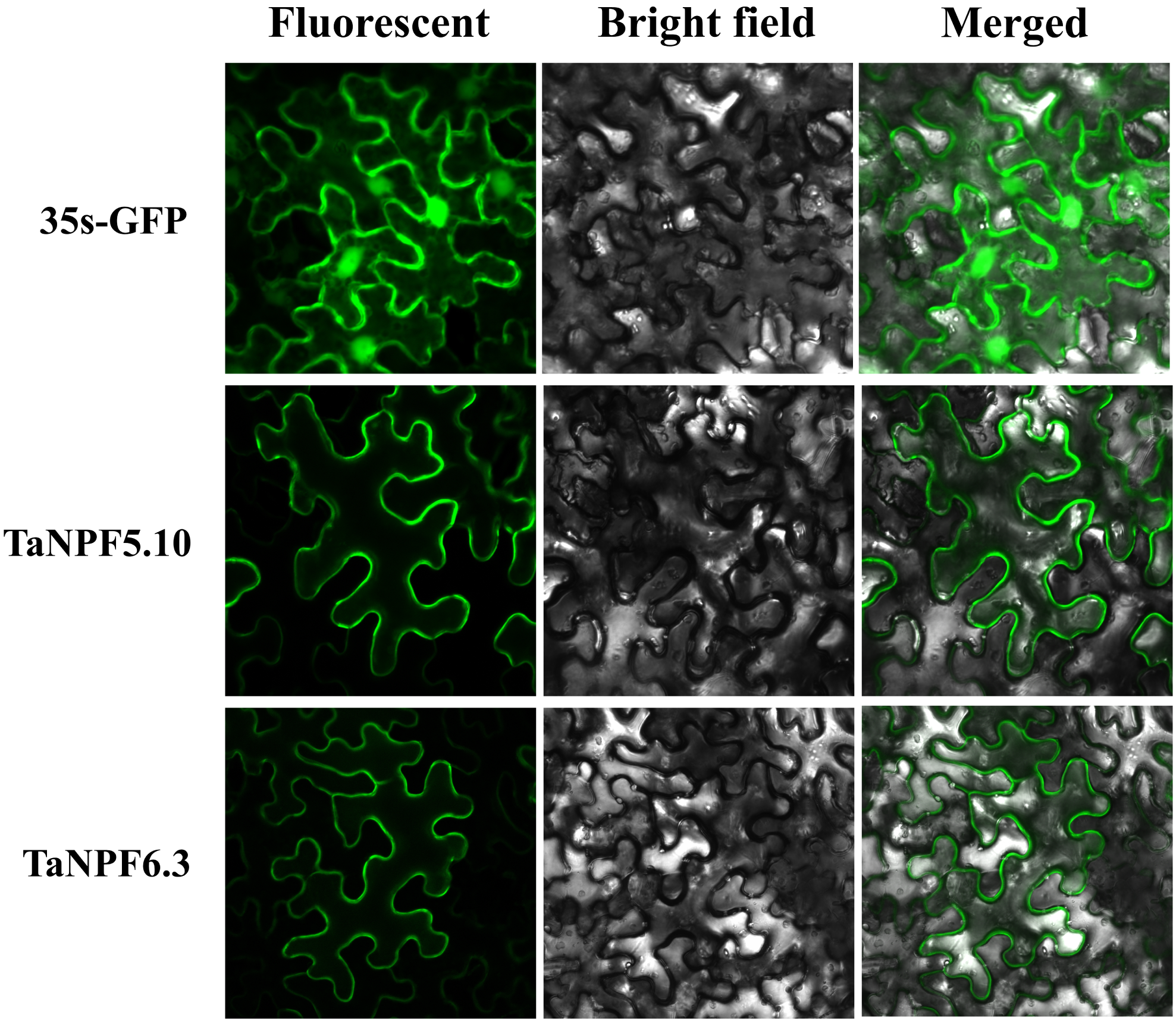

Supplement: Supplementary Figure 9 — Subcellular localization of TaNPF5.10 and TaNPF6.3 in tobacco leaves. 35S-GFP acted as the control. [file Image_9.tif]
