# Supplementary material for: Wheat potassium transporter TaHAK13 mediates K+ absorption and maintains potassium homeostasis under low potassium stress
Source: Front Plant Sci. 2022 Dec 23;13:1103235. doi: 10.3389/fpls.2022.1103235 (PMC9816385; doi:10.3389/fpls.2022.1103235)
Supplement: Supplementary Table 1 — Improved Hogland nutrient solution. Note: (1) Low potassium stress: the final concentration of K+ was 1 × 10^-5^mol·L-1, and K2SO4 was used to add K+. (2) Salt stress: NaCl with final concentration of 200 mM was added to the above nutrient solution. (3) Drought stress (20% PEG6000): PEG6000 with a final concentration of 20% was added to the above nutrient solution. [file Table_1.docx]

Table S1

| Solution component | Final concentration (mol∙L^-1^) |
| --- | --- |
| K_2_SO_4_  MgSO_4_∙7H_2_O  CaCl_2_  Ca (NO_3_)_2_∙4H_2_O  NaH_2_PO_4_∙2H_2_O  NH_4_NO_3_  H_3_BO_3_  (NH_4_)_6_Mo_7_O_24_∙4H_2_O  CuSO_4_∙5H_2_O  ZnSO_4_∙7H_2_O  MnSO_4_∙H_2_O  Fe-Na-EDTA | 1.0×10^-3^  1.0×10^-3^  1.5×10^-3^  1.0×10^-3^  2.5×10^-4^  1.0×10^-3^  1.0×10^-6^  5.0×10^-9^  5.0×10^-7^  1.0×10^-6^  1.0×10^-6^  1.0×10^-4^ |

Table S2

| **Primer names** | **Base sequence (5'- 3')** | **Restriction enzyme cutting sites** | **Purpose** |
| --- | --- | --- | --- |
| TaHAK13-F | GACCGATCTAACCGTGGGC | No | Cloning of TaHAK13 |
| TaHAK13-R | GACGGCTTGCTATGTTGATTT | No |  |
| TaHAK13-p416-F | TTAGTTTCGACGGATTCTAGAATGGATGTGGAGGGCGC | *Xba* Ⅰ | Yeast complementation |
| TaHAK13-p416-R | TTCCTGCAGCCCGGGGGATCCTCACACCCTGTAAACCATGC | *Bam*H Ⅰ |  |
| TaHAK13-GFP-F | GAGAACACGGGGGACGAGCTCATGGATGTGGAGGGCGC | *Sac* Ⅰ | Subcellular localization |
| TaHAK13-GFP-R | GGTGTCGACTCTAGAGGATCCCACCCTGTAAACCATGCCG | *Bam*H Ⅰ |  |
| pTaHAK13-1304-F | GACCTGCAGGCATGCAAGCTTATGAATCTTTACCTCTCGACCATT | *Nco* Ⅰ | GUS staining assay |
| pTaHAK13-1304-R | TACTAGTCAGATCTACCATGGAGAGAAGGCGCCAAATGCT | *Hin*d III |  |
| TaHAK13-pBT3-N-F | ATCGAATTCCTGCAGGGCCATTACGGCC ATGGATGTGGAGGGCGC | *Sfi* Ⅰ | Membrane-based yeast two-hybrid assay |
| TaHAK13-pBT3-N-R | AGCTACTTACCATGGGGCCGAGGCGGCCTCACACCCTGTAAACCATGC | *Sfi* Ⅰ |  |
| TaNPF5.10-pPR3-N-F | GTATCAACGCAGAGTGGCCATTACGGCCATGGAGGCGGAGGCCG | *Sfi* Ⅰ |  |
| TaNPF5.10-pPR3-N-R | ACATGTTTTTTCCCGGGCCGTAATGGCCTCACTGCACTGAAACACCCTTTT | *Sfi* Ⅰ |  |
| TaNPF6.3-pPR3-N-F | GTATCAACGCAGAGTGGCCATTACGGCCATGGTCGGCCTTCTCCCC | *Sfi* Ⅰ |  |
| TaNPF6.3-pPR3-N-R | ACATGTTTTTTCCCGGGCCGTAATGGCCTCAGTGGAGGCACGACTCG | *Sfi* Ⅰ |  |
| pPR3-N-F | GTCGAAAATTCAAGACAAGG | No |  |
| pPR3-N-R | AAGCGTGACATAACTAATTAC | No |  |
| TaHAK13-1300-F | ATACACCAAATCGACTCTAGAATGGATGTGGAGGGCGC | *Xba* Ⅰ | The expression of TaHAK13 in Arabidopsis |
| TaHAK13-1300-R | CGATCGGGGAAATTCGAGCTCTCACACCCTGTAAACCATGC | *Sac* Ⅰ |  |
| TaHAK13-nLUC-F | GACGAGCTCGGTACCATGGATGTGGAGGGCGC | *Kpn* I | Dual-luciferase complementation assay |
| TaHAK13-nLUC-R | CGAGATCTGGTCGACCACCCTGTAAACCATGCCG | *Sal* I |  |
| TaNRT5.10-cLUC-F | TCCCGGGGCGGTACCATGGAGGCGGAGGCCG | *Kpn* I |  |
| TaNRT5.10-cLUC-R | GCTCTGCAGGTCGACCTGCACTGAAACACCCTTTTTC | *Sal* I |  |
| TaNRT6.3-cLUC-F | TCCCGGGGCGGTACCATGGTCGGCCTTCTCCCC | *Kpn* I |  |
| TaNRT5.10-cLUC-R | GCTCTGCAGGTCGACGTGGAGGCACGACTCGGC | *Sal* I |  |
| TaActin-F | GTGTCGCACCAGAGGATCAT | No | RT-qPCR |
| TaActin-R | CGCTGGCATACAAGGACAGA | No |  |
| TaHAK13-qPCR-F | TCGCTGGGAGGGATTGT | No |  |
| TaHAK13-qPCR-R | CTGGCTTATGATGGAGAACG | No |  |

Table S3

| **Cis element** | **Sequence** | **Function** | **Position from ATG** | | **No．** |
| --- | --- | --- | --- | --- | --- |
| ABRE | CACGTG | Cis-acting element involved in the abscisic acid responsiveness | -1752，-1753，-2186 | | 3 |
| ACE | GACACGTATG | Cis-acting element involved in light responsiveness | -2183 | | 1 |
| ARE | AAACCA | Cis-acting regulatory element essential for the anaerobic induction | -1518，-1909 | | 2 |
| Box 4 | ATTAAT | Part of a conserved DNA module involved in light responsiveness | -870 | | 1 |
| CAAT-box | CAAT | Common cis-acting element in promoter and enhancer regions | -83，-104，-183，-189，-190，-214，-338，-408，-480,-500，-661，-747，-877,-938，-971，-979，-987，-1103，-1188,-1386，-1394，-1413，-1414，-1433，-1445，-1446，-1532，-1821，-1931，-1932，-1992，-2048，-2095，-2103，-2104，-2150，-2158，-2234 | | 38 |
| CAT-box | GCCACT | Cis-acting regulatory element related to meristem expression | -1253，-1791 | | 2 |
| CGTCA-motif | CGTCA | Cis-acting regulatory element involved in the MeJA-responsiveness | -35，-109，-565，-974，-2176 | | 5 |
| ERE | ATTTTAAA | Ethylene-responsive element | -1892 | | 1 |
| G-Box | CACGTG | Cis-acting regulatory element involved in light responsiveness | -1752 | | 1 |
| G-box | TACGTG | Cis-acting regulatory element involved in light responsiveness | -2185 | | 1 |
| GT1-motif | GGTTAA | Light responsive element | -1947，-2030，-2031 | | 3 |
| MBS | CAACTG | MYB binding site involved in drought-inducibility | -1705 | | 1 |
| MRE | AACCTAA | MYB binding site involved in light responsiveness | -1971 | | 1 |
| Root motif TAPOX1 | ATAAT | Root-specific expression-related cis-acting element | -216，-230，-968，-1120，-1360 | | 5 |
| TATA-box | TATA | Core promoter element around -30 of transcription start | -3，-422，-423，-542，-543，-544，-635，-637，-1035，-1036，-1037，1038，-1081，-1098，-1100，-1416，-1417，-1418，-1448，-1449，-2058 | | 21 |
| TC-rich repeats | GTTTTCTTAC | Cis-acting element involved in defense and stress responsiveness | | -1298，-1983 | 2 |
| TCT-motif | TCTTAC | Part of a light responsive element | -1987 | | 1 |
| TGA-element | AACGAC | Auxin-responsive element | -1833 | | 1 |
| W box | TTGACC | WRKY identifies and binds elements | -180，-248 | | 2 |

Table S4

| ID | Homologous protein | cDNA length/bp | Function |
| --- | --- | --- | --- |
| 1 | TaPRB1-2 | 891 | Triticum aestivum pathogenesis-related protein PRB1-2 |
| 2 | TaIAA3 | 1495 | Triticum aestivum auxin-responsive protein IAA3 |
| 3 | TaNPF5.10 | 1834 | Triticum aestivum protein NRT1/ PTR FAMILY 5.10 |
| 4 | TaDDR48 | 1272 | Triticum aestivum stress protein DDR48 |
| 5 | TaNPF6.3 | 2373 | Triticum aestivum protein NRT1/ PTR FAMILY 6.3 |
